# Meis1 Targets Protein Tyrosine Phosphatase Receptor J in Fibroblast to Retard Chronic Kidney Disease Progression

**DOI:** 10.1002/advs.202309754

**Published:** 2024-08-20

**Authors:** Mi Bai, Shuang Xu, Mingzhu Jiang, Yuxian Guo, Dandan Hu, Jia He, Ting Wang, Yu Zhang, Yan Guo, Yue Zhang, Songming Huang, Zhanjun Jia, Aihua Zhang

**Affiliations:** ^1^ Department of Nephrology, State Key Laboratory of Reproductive Medicine Children's Hospital of Nanjing Medical University Nanjing 210008 China; ^2^ Jiangsu Key Laboratory of Early Development and Chronic Diseases Prevention in Children Nanjing Medical University Nanjing 210029 China; ^3^ Nanjing Key Lab of Pediatrics Children's Hospital of Nanjing Medical University Nanjing 210008 China; ^4^ Medical School of Nanjing University Nanjing 210093 China

**Keywords:** chronic kidney disease, fibroblast, Meis1, Ptprj, renal fibrosis

## Abstract

Renal fibrosis is a common pathological feature of chronic kidney disease (CKD) with the proliferation and activation of myofibroblasts being definite effectors and drivers. Here, increased expression of Meis1 (myeloid ecotropic viral integration site 1) is observed, predominantly in the nucleus of the kidney of CKD patients and mice, and negatively correlates with serum creatinine. Fibroblast‐specific knock‐in of Meis1 inhibits myofibroblast activation and attenuates renal fibrosis and kidney dysfunction in CKD models. Overexpression of Meis1 in NRK‐49F cells suppresses the pro‐fibrotic response induced by transforming growth factor‐β1 but accelerates by its knockdown. Mechanistically, Meis1 targets protein tyrosine phosphatase receptor J (Ptprj) to block renal fibrosis by inhibiting the proliferation and activation of fibroblasts. Finally, a new activator of Ptprj is identified through computer‐aided virtual screening, which has the effect of alleviating renal fibrosis. Collectively, these results illustrate that the Meis1/Ptprj axis has therapeutic potential for clinically treating CKD.

## Graphical abstract

Myeloid ecotropic viral integration site 1 (Meis1) is elevated in CKD patients and negatively correlated with serum creatinine. Overexpression of Meis1 in fibroblasts alleviates renal fibrosis by targeting protein tyrosine phosphatase receptor J (Ptprj) to inhibit fibroblast proliferation and activation. Activation of the Meis1/Ptprj axis has therapeutic potential for treating CKD.

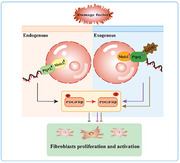

## 1 Introduction

Chronic kidney disease (CKD) is the progressive loss of kidney function over many years, can arise via multiple heterogeneous disease pathways, and has become a major global health problem.^[^ ^1^ ^]^ Current measures aimed at controlling the underlying causes of CKD, such as diet and medications, have not been sufficient to prevent the progression of CKD to end‐stage renal disease (ESRD). Therefore, it is imperative to investigate the pathophysiological mechanism of CKD and develop new therapeutic strategies to slow, and hopefully even reverse, CKD.

Regardless of the etiological cause of CKD, the most common pathological feature is renal fibrosis, which currently is the best predictor of progression and irreversibility of all types of CKD.^[^ ^2^ ^]^ Renal fibrosis is characterized by excessive deposition of extracellular matrix (ECM), leading to tissue remodelling and scar formation of renal parenchyma. Although renal injury that develops under pathological conditions usually initiates from tubular epithelial cells (TECs), it is inevitable that all the renal responses converge as a common result, that being the proliferation and activation of myofibroblasts.^[^ ^3^ ^]^ Studies over the past decade have shown that myofibroblasts, the main collagen‐producing cells, are the definite effectors and drivers of renal fibrosis. Accordingly, suppressing the activation and accumulation of myofibroblasts and deposition of ECM is an effective strategy to delay the progression of fibrosis. Recently, Kramann et al. used single‐cell RNA sequencing (scRNA‐seq) and parabiosis models to demonstrate that resident fibroblasts are the main precursors of myofibroblasts in renal fibrosis.^[^ ^4^ ^]^ Therefore, targeting interstitial fibroblasts as a precise molecular intervention is of great interest toward delaying the progression of renal fibrosis and improving CKD outcomes.

Kidney stroma plays an indispensable role in renal fibrosis, but little is currently known regarding transcriptional regulators of the renal interstitium. Myeloid ecotropic viral integration site 1 (Meis1) is a member of the three amino acid loop extension homeodomain transcription factor family, which has been intensively studied in haematopoiesis and leukaemia,^[^ ^5^ ^]^ cardiac regeneration,^[^ ^6^ ^]^ cancer,^[^ ^7^ ^]^ and embryonic development.^[^ ^8^ ^]^ Through transcriptional control of downstream targets, Meis1 is involved in metabolic regulation,^[^ ^9^ ^]^ reactive oxygen species (ROS) production,^[^ ^10^ ^]^ as well as other biological processes. Several lines of inquiry have demonstrated that overexpression of Meis1 in cardiomyocytes significantly restricts cardiomyocyte proliferation and heart regeneration in mice after myocardial infarction.^[^ ^6^, ^11^ ^]^ Meis1 has also been reported to be expressed in the kidney interstitium during nephrogenesis with Hisa et al. observing kidney development defects in Meis1‐mutant mouse embryos.^[^ ^12^ ^]^ Moreover, a previous study showed that Meis1 overexpression significantly inhibits proliferation, invasion, and migration of renal carcinoma cells.^[^ ^13^ ^]^ However, the role of Meis1 in renal fibrosis and CKD has not been determined.

To investigate the primary molecular mechanism of regulating the gene expression of Meis1, we established a unilateral ureteral obstruction (UUO) model using 8–12 wk‐old male transgenic mice and renal fibroblasts with a conditional knock‐in (cKI) of the Meis1 gene. Kidney tissues from the model mice were harvested for RNA sequencing (RNA‐seq) analysis. The RNA‐seq results were compared against the Gene Transcription Regulation Database (GTRD, http://gtrd.biouml.org/) and multiple Meis1 binding sites were detected in the promoter region of protein tyrosine phosphatase receptor J (Ptprj). This suggests that Ptprj may be a downstream regulatory target of Meis1.

Ptprj is a member of the receptor‐type protein tyrosine phosphatase family, has been implicated in various signal pathways, and is known for its anti‐proliferation and anti‐tumor functions.^[^ ^14^ ^]^ Ptprj dephosphorylates several candidate substrates, including platelet‐derived growth factor receptor (PDGFRβ),^[^ ^15^ ^]^ hepatocyte growth factor receptor (HGFR),^[^ ^16^ ^]^ and vascular endothelial growth factor receptor (VEGFR),^[^ ^17^ ^]^ among others. The dephosphorylation by Ptprj negatively interferes with cell proliferation and migration.^[^ ^18^ ^]^ Multiple pathways are involved in fibrosis with platelet‐derived growth factor (PDGF)‐signaling being one of the central mediators.^[^ ^19^ ^]^ PDGFRβ is expressed in stromal mesenchymal cells and upon phosphorylation activation, it modulates various downstream signaling pathways, such as phosphatidyl inositol 3‐kinase (PI3K) and Janus kinase, etc., driving the cell proliferation and migration and production of ECM. This ultimately leads to the development of multi‐organ fibrosis, including renal fibrosis.^[^ ^19^, ^20^ ^]^ Ptprj is able to negatively regulate the level of PDGFRβ phosphorylation. Accordingly, it is not difficult to speculate that Meis1‐control of Ptprj may be an important component of renal fibrosis, an aspect that has not been previously appreciated.

In the current study, we evaluated human renal biopsy specimens and used a variety of CKD animal models, as well as renal fibroblasts, to investigate whether Meis1/Ptprj signaling could restrain the proliferation and activation of fibroblasts and improve renal fibrosis, thereby blunting the progression of CKD. Finally, we validated the results by demonstrating that the pharmacological activator of Ptprj could attenuate renal fibrosis. The findings should provide valuable insight into potential new methods for the treatment of patients with CKD.

## 2 Results

### 2.1 Meis1 is Increased in Fibrotic Kidneys of CKD Patients and Mice

**Figure 1:**
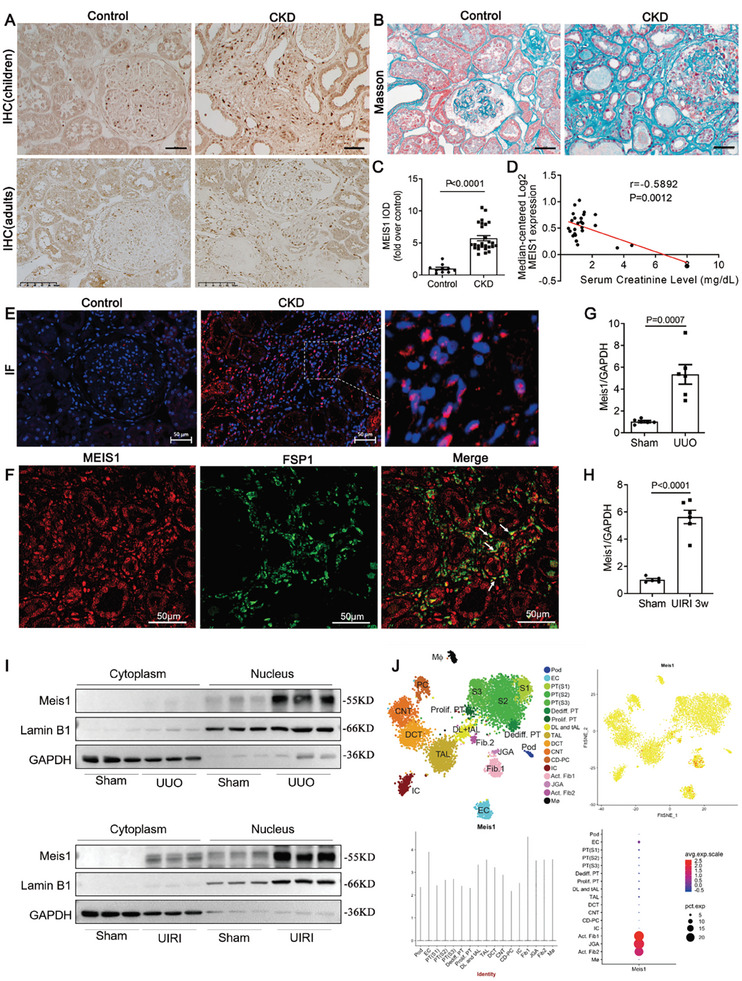
Meis1 is increased in fibrotic kidneys of CKD patients and mice. A) Representative micrographs of MEIS1 expression in children and adults with CKD (*n *= 26) through immunohistochemical staining (×200). Normal kidney tissues were collected from patients without proteinuria who underwent partial nephrectomy for benign kidney tumors (*n *= 10). B) Representative images of Masson trichrome staining of renal tissue in CKD patients (*n *= 17) and normal control (*n *= 4). Scale bar, 50 µm. C) Quantitative data of MEIS1 expression in children and adults with CKD (*n *= 10 in Control group, *n *= 26 in CKD group). D) Pearson's r correlation analysis between serum creatinine and MEIS1 expression in CKD patients (*n *= 27) from NephroSeq database (https://nephroseq.com). E) Images are representative of MEIS1 immunofluorescence staining in normal and CKD patients (*n *= 9). Scale bar, 50 µm. F) Immunofluorescence co‐localization analysis of MEIS1 and FSP1 in human kidney tissue. Scale bar: 50 µm. G,H) Quantitative determination of renal Meis1 mRNA levels by qRT‐PCR in UUO and UIRI models (*n *= 6). I) Western blotting analysis of renal Meis1 in cytoplasm and nucleus in UUO and UIRI models. Lamin B1 and GAPDH was used as loading control (*n *= 3 in each group). J) The expression of Meis1 in the Kidney Interactive Transcriptomics database (Wu et al, 2019). Data are presented as mean ± SEM. Data were statistically analyzed using unpaired Two‐tailed Student's t‐test (C, G, and H) or Pearson's r correlation analysis (D). The P‐values were shown in the figures. Abbreviations: CKD, chronic kidney disease; FSP1, fibroblast‐specific protein 1; UUO, unilateral ureteral obstruction; UIRI, unilateral ischemia/reperfusion injury.

To validate the potential role of Meis1 in kidney disease, we performed immunohistochemical and immunofluorescence staining. MEIS1 was expressed at low levels in the control kidney sections, but significantly increased in the interstitium of children and adult CKD patients with varying degrees of fibrosis (**Figure** **1**A–C). The levels of MEIS1 negatively correlated with serum creatinine in CKD patients (*n* = 27) from NephroSeq database (https://nephroseq.com) (r = −0.5892, P = 0.0012; Figure 1D). Immunofluorescence staining showed that increased MEIS1 expression in CKD patients was mainly in the nucleus (Figure 1E) and could be co‐located with FSP1 (a marker of fibroblasts) (Figure 1F). There was also a small amount of Meis1 expression in the cytoplasm. We then evaluated the renal expression of Meis1 using two CKD mouse models, a unilateral ureteral obstruction (UUO) model and a unilateral ischemia/reperfusion injury (UIRI) model. We found that Meis1 expression increased significantly in the kidney cortex of animals from both these CKD mouse models (Figure 1G,H), with the level of expression being much greater in the nuclei compared to that in the cytoplasm (Figure 1I). We also queried the single‐cell RNA‐Seq data sets for kidney disease available in the Kidney Interactive Transcriptomics database (http://humphreyslab.com/SingleCell/). Meis1 is expressed in multiple cell populations of the kidneys of mice deposited in Mouse UUO d14 dataset^[^ ^21^ ^]^ with its expression being more pronounced in activated fibroblasts (Figure 1J).

### 2.2 Fibroblast‐Specific Overexpression of Meis1 Ameliorated Renal Fibrosis After Induction of UUO, UIRI, and FA

Considering the necessity of Meis1 expression in the interstitium during renal development and the importance of fibroblasts in the occurrence and development of renal fibrosis, we generated fibroblast‐specific Meis1 conditional knock‐in (Meis1‐cKI) mice (Figure S1A, Supporting Information). Cultured primary fibroblasts from these mice, which confirmed by cell morphology and fibroblast‐specific protein 1 (Fsp1) immunofluorescence staining (Figure S1B, Supporting Information), exhibited a significant increase in Meis1 expression (Figure S1C, Supporting Information); however, there was no increase in Meis1 expression in cultured proximal tubular cells compared with those from wide type (WT) mice (Figure S1D, Supporting Information).

**Figure 2:**
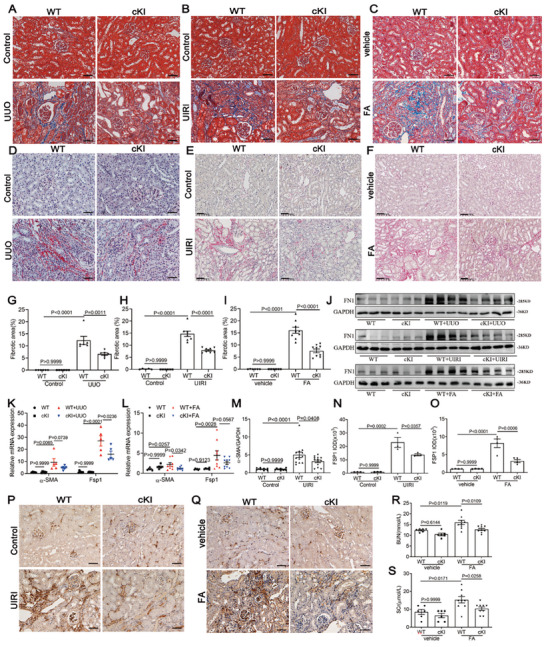
Fibroblast‐specific overexpression of Meis1 ameliorated renal fibrosis in CKD models. A–C) Photomicrographs illustrating Masson trichrome staining (blue) staining of renal tissue (×200) in WT and cKI mice after UUO, UIRI or FA model. Scale bar, 50 µm. In UUO model, *n *= 6 in each group. In UIRI model, *n *= 5 or 6 in Sham group and *n *= 8 in UIRI group. In FA model, *n *= 6 in vehicle group and *n *= 9 in FA group. D–F) Representative images for Picrosirius red staining (×200) in WT and cKI mice after UUO, UIRI or FA model. Scale bar, 50 µm. G–I) Quantitative data of fibrosis area according to Masson staining. J) Western blotting analysis of FN1 levels in WT and cKI mice after UUO, UIRI, or FA model (*n *= 3 or 4 in each group). K,L) qRT‐PCR analysis of α‐SMA and Fsp1 mRNA expressions in WT and cKI mice after UUO (*n *= 5 in WT group, *n *= 6 in cKI group) or FA model (*n *= 6 in vehicle group, *n *= 9 in FA group). M) qRT‐PCR analysis of renal α‐SMA mRNA expression in UIRI model (*n *= 11 in WT group, *n *= 13 in cKI group). N–Q) Representative micrographs and quantification showed Fsp1 expression through immunohistochemical staining (×200) in WT and cKI mice after UIRI or FA model (*n *= 3 or 4 in each group). Scale bar, 50 µm. R,S) Scr and BUN concentrations were measured in FA‐induced CKD WT or cKI mice (*n *= 6 in vehicle group, *n *= 9 in FA group). Data are presented as mean ± SEM. Statistical analysis was performed using One‐way ANOVA followed by Bonferroni. The P‐values were shown in the figures. Abbreviations: UUO, unilateral ureteral obstruction; UIRI, unilateral ischemia/reperfusion injury; FA, folic acid; BUN, blood urea nitrogen; Scr, serum creatinine.

We then established the UUO, UIRI, and FA models using the Meis1‐cKI and WT mice. Specific overexpression of Meis1 in fibroblasts significantly attenuated the degree of renal interstitial fibrosis, as determined by Masson's trichrome (**Figure** **2**A–C,G–I) and Picrosirus Red staining (Figure 2D–F) and the deposition of extracellular matrix components FN1, Collagen I, and Collagen III as measured at both the mRNA level (Figure S2A–C, Supporting Information). The FN1 protein levels in three models also inhibited in fibroblast‐specific Meis1‐cKI mice (Figure 2J; Figure S1D–F, Supporting Information). We also assessed mice from our models for Fsp1 and α‐SMA levels, which are considered to be reliable markers for the expansion and activation of fibroblasts into myofibroblasts in tissues. We found that specific overexpression of Meis1 in fibroblasts significantly blunted the up‐regulation of Fsp1 and α‐SMA in both UUO mice (Figure 2K), FA mice (Figure 2L) and UIRI mice (Figure 2M) at mRNA levels. The immunohistochemical staining of Fsp1 in kidney tissue also confirmed this (Figure 2N–Q). Exogenous overexpression of Meis1 through high‐pressure injection of the plasmid in the tail vein also showed that enhanced Meis1 could improve renal fibrosis induced by UUO or UIRI (Figure S3, Supporting Information). These results revealed that the overexpression of Meis1 in fibroblasts dampened the proliferation and activation of fibroblasts, thereby significantly reducing ECM deposition and renal fibrosis.

The FA‐induced renal injury model is well‐characterized animal model of renal tubulointerstitial fibrosis. Compared to UUO and UIRI models, which lack functional readouts, renal function in the FA model can be assessed as a measure of CKD.^[^ ^22^ ^]^ As shown in Figure 2R,S, there were no significant differences in serum creatinine (Scr) or blood urea nitrogen (BUN) levels between the WT and Meis1‐cKI mice at baseline. After 4 wk of folic acid treatment, there was a slight statistically significant increase in Scr and BUN levels; however, these increases were reversed with Meis1 overexpression in fibroblasts. Collectively, these results demonstrate that Meis1 overexpression in fibroblasts improved renal function that was induced by FA.

### 2.3 Meis1 Inhibited TGF‐β1‐Induced Proliferation and Activation of Rat Kidney‐49F (NRK‐49F) Cells

**Figure 3:**
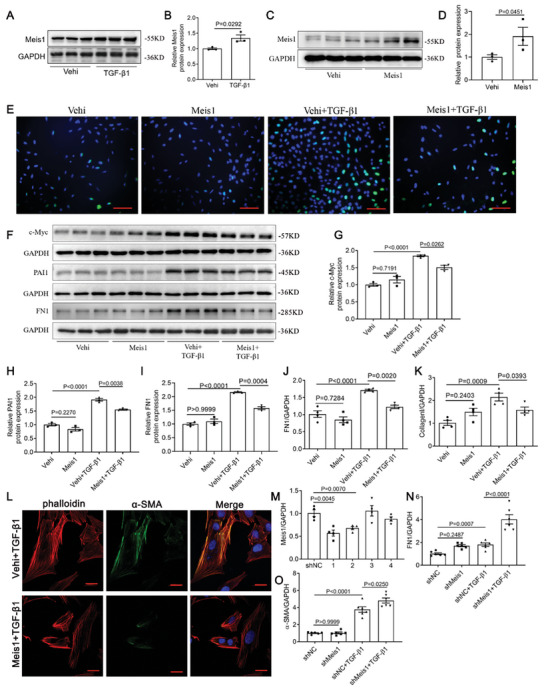
Meis1 inhibited TGF‐β1‐induced proliferation and activation of NRK‐49F cells. A,B) Western blotting analysis of Meis1 after TGF‐β1 treatment (*n *= 3). C,D) Western blotting analysis of Meis1 after transfected with Meis1 plasmids (*n *= 3). In figure E–L, NRK‐49F cells were transfected with vector or Meis1 plasmids, and then treated with TGF‐β1 (10 ng mL^−1^) for 24 or 48 h. E) Immunofluorescence images of EdU (*n *= 3). Scale bar, 50 µm. F) Western blotting for PAI1, c‐Myc and FN1 (*n *= 3). G–I) Semi‐quantitative analysis for PAI1, c‐Myc and FN1. J,K) qRT‐PCR analysis of FN1 and Collagen I mRNA expressions (*n *= 4). L) Immunofluorescence co‐localization analysis of α‐SMA and phalloidin (*n *= 3). Scale bar, 20 µm. In figure M–O, NRK‐49F cells were transfected with shNC or shMeis1 plasmids, and then treated with TGF‐β1 (10 ng mL^−1^) for 24 h. M) mRNA levels of Meis1 after shRNA transfection (*n *= 4). N,O) qRT‐PCR analysis of FN1 (*n *= 6) and α‐SMA (*n *= 5) mRNA expressions. Data are presented as mean ± SEM. Data were statistically analyzed using unpaired Two‐tailed Student's t‐test (B,D) or One‐way ANOVA followed by Bonferroni (G–K and M–O). The P‐values were shown in the figures. Abbreviations: TGF‐β1, transforming growth factor‐β1; Vehi, vehicle; PAI1, plasminogen activator inhibitor 1.

To further corroborate the specific role of Meis1 in fibroblasts, cultured NRK‐49F cells were transfected with Meis1 overexpression plasmids or Meis1 shRNA plasmids and then stimulated with TGF‐β1. Exposure of NRK‐49F cells to TGF‐β1 induced Meis1 expression (**Figure** **3**A,B). After overexpression Meis1 (Figure 3C,D), the activated renal fibroblasts induced by TGF‐β1 treatment were attenuated, as evidenced by the proliferation indicators EdU staining (Figure 3E), protein levels of c‐Myc and PAI1 (Figure 3F–H), as well as the mRNA and protein levels of fibroblast‐related molecules FN1, Collagen I, and α‐SMA (Figure 3I–L). Consistent with these results, inhibition of Meis1 expression with shRNA resulted in increased TGF‐β1‐induced expression of FN1 and α‐SMA (Figure 3M–O).

### 2.4 Ptprj is the Transcriptional Target of Meis1 in Kidneys

**Figure 4:**
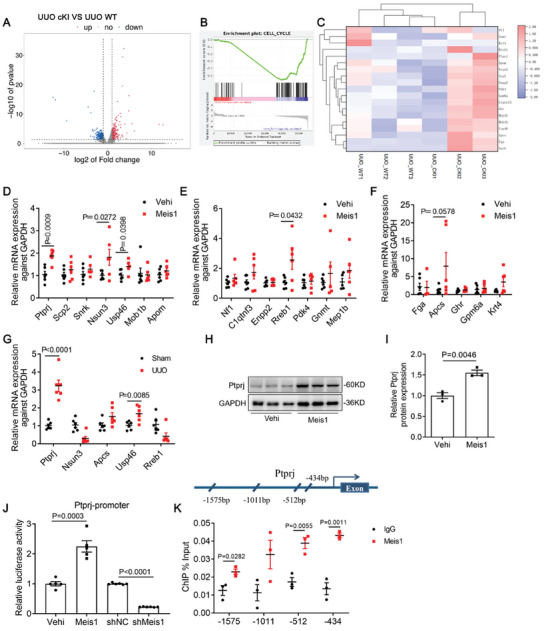
Ptprj is the transcriptional target of Meis1 in kidneys. A) Volcano plot for differentially expressed genes in the kidneys from WT and cKI UUO mice based on RNA sequencing analysis (up‐regulated ≥ 2.0‐fold or down‐regulated ≤ 0.5‐fold, *P* < 0.05). B) GSEA for the phenotype ontology term”cell cycle” in wild‐type and WT and cKI mice after UUO model. C) Heatmap of genes associated with proliferation and differentiation. D–F) Validation mRNA expression of up‐regulated genes in NRK‐49F cells after Meis1 overexpression (*n *= 6). G) Validation mRNA expression of Ptprj, Nsun3, Apcs, Usp46 and Rreb1 in UUO model (*n *= 6). H,I) Western blotting analysis of Ptprj after Meis1 overexpression (*n *= 3). J) Luciferase assay of NRK‐49F cells transfected with the pGL4.19‐Ptprj promoter reporters with Meis1 (*n *= 5) or shMeis1 (*n *= 6) plasmids. K) ChIP analysis of Meis1 at the regions of the Ptprj promoter (*n *= 3). Data are presented as mean ± SEM. Statistical analysis was performed using Two‐tailed Student's t‐test. The P‐values were shown in the figures. Abbreviations: Vehi, vehicle; UUO, unilateral ureteral obstruction; ChIP, chromatin immunoprecipitation assay.

To investigate the downstream mechanism of Meis1, we performed RNA sequencing analysis and assessed differentially expressed genes (DEGs) in the renal cortex of WT and Meis1‐cKI mice after UUO induction. Compared to that of the WT UUO mice, the Meis1‐cKI UUO mice exhibited 288 up‐regulated DEGs (up‐regulated ≥ 2.0‐fold, *p* < 0.05; **Figure** **4**A). Because gene set enrichment analysis (GSEA) showed Meis1 overexpression inhibited the phenotype ontology term “cell cycle” (Figure 4B), we retrieved the functions of the DEGs to identify the genes related to fibrosis and cell proliferation. A total of 19 genes were identified as potential matches (Figure 4C). Next, qRT‐PCR was used to verify the up‐regulated expression of the identified genes in Meis1‐overexpessing NRK‐49F cells (Figure 4D–F) and confirmed the results in UUO model (Figure 4G). Ultimately, we determined that *Ptprj* was a potential target gene of Meis1. The protein levels of Ptprj were significantly increased in NRK‐49F cells overexpressing Meis1 (Figure 4H,I). We then co‐transfected NRK‐49F cells with a 2‐kb‐long pGL4.19‐Ptprj reporter gene and Meis1 or shMeis1‐expressing plasmids and performed luciferase assays. As shown in Figure 4J, pGL4.19‐Ptprj was activated by Meis1 and inhibited after knockdown of Meis1 in NRK‐49F cells. We further confirm the effect of Meis1 on regulating *Ptprj* expression using a chromatin immunoprecipitation (ChIP) assay (Figure 4K).

### 2.5 The Function of Ptprj in NRK‐49F Cell and UUO Model

**Figure 5:**
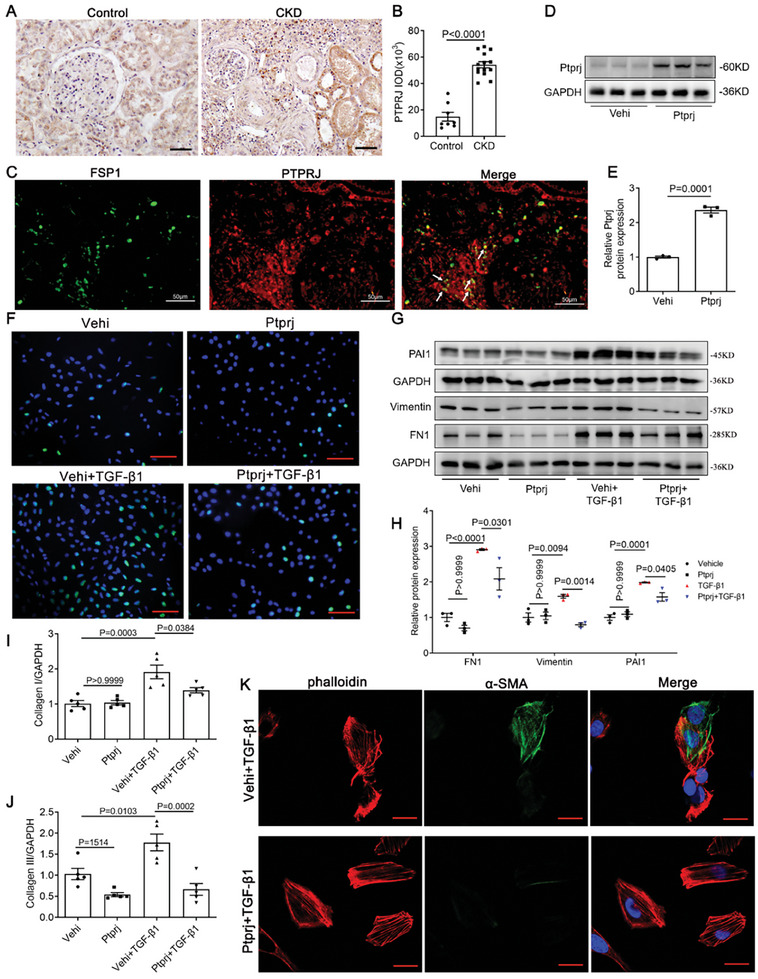
Ptprj is increased in CKD patients and suppressed TGF‐β1‐induced proliferation and activation of NRK‐49F cells. A,B) Representative images and quantitative data of PTPRJ expression in children with CKD (*n *= 14) and normal kidney tissues (*n *= 8) through immunohistochemical staining. Scale bar, 50 µm. C) Immunofluorescence co‐localization analysis of PTPRJ and FSP1 in human kidney tissue. Scale bar: 50 µm. In figure D–M, NRK‐49F cells were transfected with vector or Ptprj plasmids, and then treated with TGF‐β1 (10 ng mL^−1^) for 24 or 48 h. D,E) Western blotting analysis of Ptprj after Ptprj overexpression in NRK‐49F cells (*n *= 3). F) Immunofluorescence images of EdU (*n *= 3). Scale bar, 50 µm. G,H) Western blotting for PAI1, FN1, and Vimentin protein expressions (*n *= 3 in each group). I,J) Collagen I and Collagen III mRNA expressions (*n *= 5). K) Immunofluorescence co‐localization analysis of α‐SMA and phalloidin (*n *= 3). Scale bar, 20 µm. Data are presented as mean ± SEM. Data were statistically analyzed using unpaired Two‐tailed Student's t‐test (B,E) or One‐way ANOVA followed by Bonferroni (H–J). The P‐values were shown in the figures. Abbreviations: CKD, chronic kidney disease; FSP1, fibroblast‐specific protein 1; TGF‐β1, transforming growth factor‐β1; Vehi, vehicle; PAI1, plasminogen activator inhibitor 1.

To determine the role of Ptprj in kidney disease, we performed immunohistochemical staining. PTPRJ was expressed at low levels in kidney sections but increased significantly in the renal tubular cells and the interstitium of CKD patients (**Figure** **5**A,B). We confirmed the increased expression in by interstitium the double staining of PTPRJ and FSP1 (Figure 5C). Consistent with the function of Meis1, overexpression of Ptprj (Figure 5D,E) inhibited the proliferation and activation evidenced by EdU staining (Figure 5F), protein levels of PAI1 (Figure 5G,H), as well as the mRNA levels of fibroblast‐related molecules Collagen I, Collagen III (Figure 5I,J) and protein levels of FN1, Vimentin (Figure 5G,H) and α‐SMA (Figure 5K).

**Figure 6:**
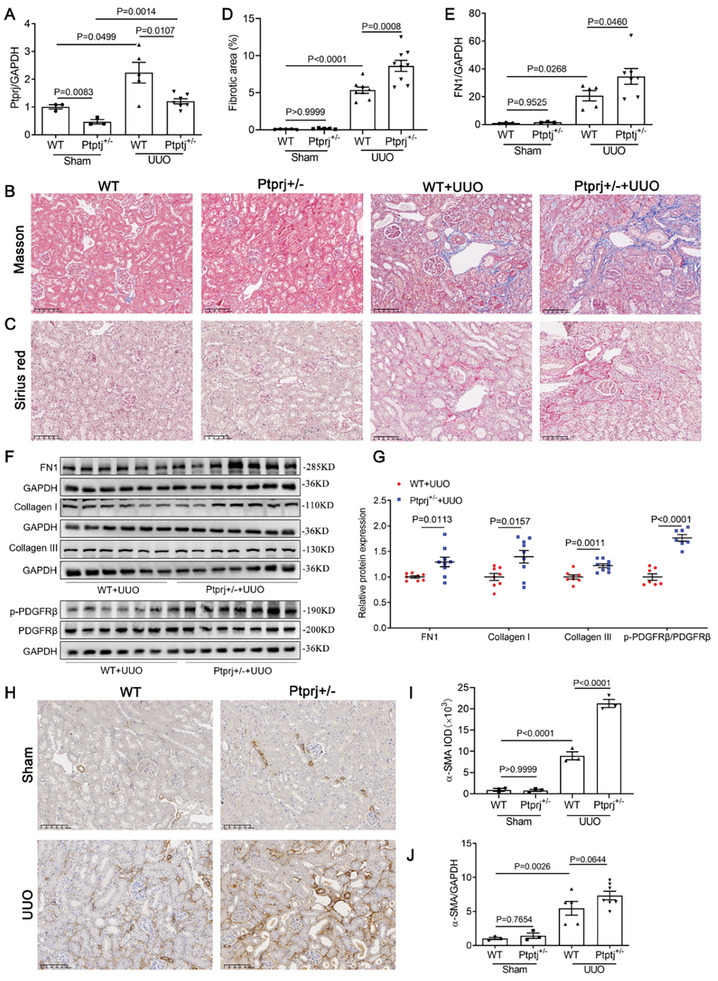
Ptprj heterozygous knockout aggravated UUO‐induced renal fibrosis. A) qRT‐PCR analysis of Ptprj mRNA expression (*n *= 3 in Sham group, *n *= 5 in WT+UUO group, *n *= 7 in Ptprj^+/−^+UUO group. B,C) Representative images of Masson trichrome staining (blue) (×200) and Picrosirius red staining of renal tissue (×200) in WT and Ptprj^+/−^ mice after UUO model. Scale bar, 100 µm. D) Quantitative data of fibrosis area according to Masson staining (*n *= 5 in Sham group, *n *= 8 or 9in UUO group). E) qRT‐PCR analysis of FN1 mRNA expression (*n *= 3 in Sham group, *n *= 5 in WT+UUO group, *n *= 7 in Ptprj^+/−^+UUO group). F,G) The protein expressions of FN1, Collagen I, and Collagen III were analyzed by western blotting (*n *= 8 in WT+UUO group, *n *= 9 in Ptprj^+/−^+UUO group), as well as the expression of PDGFRβ and p‐PDGFRβ (*n *= 7 in each group). H,I) Representative immunohistochemical images show α‐SMA staining in kidney and quantitative analysis of α‐SMA (*n *= 3 in each group). Scale bar, 100 µm. J) qRT‐PCR analysis of α‐SMA mRNA expression (*n *= 3 in Sham group, *n *= 5 in WT+UUO group, *n *= 7 in Ptprj^+/−^+UUO group). Data are presented as mean ± SEM. Data were statistically analyzed using unpaired Two‐tailed Student's t‐test (G) or One‐way ANOVA followed by Bonferroni (A, D, E, I, and J). The P‐values were shown in the figures. Abbreviations: UUO, unilateral ureteral obstruction; PDGFRβ, platelet derived growth factor receptor β.

Considering that Ptprj homozygous mice have the possibility of embryo death,^[^ ^23^ ^]^ we generated Ptprj heterozygous knockout (*Ptprj^+/−^*) mice (**Figure** **6**A; Figure S4A,B, Supporting Information) and established a UUO model. Masson's trichrome and Sirius red staining (Figure 6B–D) revealed that kidney fibrosis induced by UUO was deteriorated in *Ptprj^+/−^* mice, compared with that in the WT group. qRT‐PCR analysis showed Ptprj knockdown promoted the deposition of extracellular matrix components FN1 expression (Figure 6E), as well as the protein levels of FN1, Collagen I and Collagen III detected by western blotting (Figure 6F,G). The protein levels of FN1, Collagen I and Collagen III had no change in WT or *Ptprj^+/−^* mice under normal conditions (Figure S4C, Supporting Information). The proliferation and activation of fibroblasts according α‐SMA expression also deteriorated after Ptprj knockdown in UUO mice (Figure 6H–J). Exogenous overexpression of Ptprj through high‐pressure injection of the plasmid in the tail vein showed that overexpression of Ptprj could improve renal fibrosis induced by UUO (Figure S5, Supporting Information). Ptprj has been reported to be a key mediator of inhibiting cell proliferation by dephosphorylation of PDGFRβ. As shown in Figure 6F,G, Ptprj knockdown increased the phosphorylation of PDGFRβ levels in fibrotic kidneys compared with that in the controls, which suggests that Ptprj may regulate renal fibrosis through PDGFRβ.

Taken together, Meis1 is likely to activate Ptprj through transcriptional activation and promote its expression, thereby reducing TGF‐β1‐induced cell damage and attenuating renal fibrosis.

### 2.6 A Novel Ptprj Activator Blunted Fibrotic Response in NRK‐49F Cells and Protected Against Renal Fibrosis in UUO and UIRI Models

**Figure 7:**
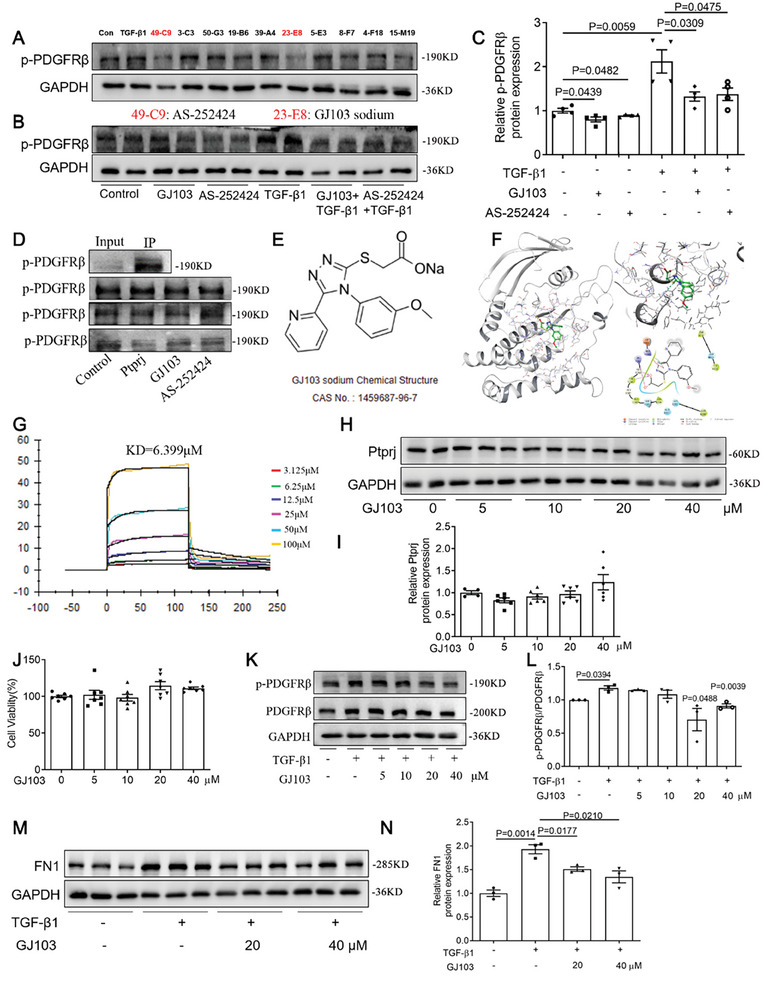
A novel Ptprj activator blunted fibrotic response in NRK‐49F cells. A) 10 high docking score molecules showed Ptprj activating effect in dephosphorylation of PDGFRβ in NRK‐49F cells, of which AS‐252424 and GJ103 sodium showed stronger activity (*n *= 2). TGF‐β1, a reported PDGFRβ phosphorylation activator, is involved as a contrast. B,C) Dephosphorylation of PDGFRβ by AS‐252424 and GJ103 sodium after TGF‐β1 stimulation in NRK‐49F cells (*n *= 3). D) Ptprj activating effect in cell‐free dephosphorylation system of three independent experiments. E) Chemical structure of GJ103 sodium. F) Proposed binding model of GJ103 sodium binding to the human PTPRJ catalytic domain. Three and 2D illustrations of the interaction between GJ103 sodium and human PTPRJ catalytic domain. G) Physical interaction between GJ103 sodium and PTPRJ performed by Surface Plasmon Resonance (SPR). H,I) Western blotting analysis of Ptprj after different concentrations of GJ103 treatment in NRK‐49F cells (*n *= 4‐6). J) CCK8 analysis of cell viability at different concentrations of GJ103 in NRK‐49F cells (*n *= 7 in each group). K,L) Western blotting analysis and quantitative data for PDGFRβ and p‐PDGFRβ in NRK‐49F cells after pre‐treatment of different concentrations of GJ103 following TGF‐β1 (*n *= 3). The P value in bar chart versus TGF‐β1 group. M,N) Western blotting analysis and quantitative data for FN1 in NRK‐49F cells after pre‐treatment different concentrations of GJ103 following TGF‐β1 (*n *= 3). Data are presented as mean ± SEM. Statistical analysis was performed using Two‐tailed Student's t‐test. The P‐values were shown in the figures. Abbreviations: PDGFRβ, platelet‐derived growth factor receptor β; TGF‐β1, transforming growth factor‐β1.

The data described above prompted us to investigate the therapeutic potential of targeting Ptprj. Therefore, we screened effective Ptprj activators by molecular docking and molecules with high docking scores were further screened by detecting the dephosphorylation activity of Ptprj on PDGFRβ. By directly stimulating NRK49F cells with 10 small molecules, we found that AS252424 and GJ103 significantly inhibited the phosphorylation level of PDGFRβ (**Figure** **7**A), which also inhibited the phosphorylation level of PDGFRβ after co‐stimulation with TGF‐β1 (Figure 7B,C). Finally, through dephosphorylation of the PDGFRβ in vitro, we found that GJ103 can better activate Ptprj (Figure 7D). As shown by the binding models displayed as 2D and 3D diagrams, GJ103 can form two conventional H‐bonds with GLY1244 and VAL1243, one salt bridge with ARG1245, as well as one Pi‐cation and one Pi‐Pi stacking (Figure 7E,F). In addition, the binding affinity of GJ103 to recombinant PTPRJ protein was quantitatively analyzed by surface plasmon resonance (SPR) equipment. The results showed that the molecular binding activity was reliable, and the binding affinity (KD value) was 6.399 µm (Figure 7G). The effect of GJ103 on Ptprj was further verified in NRK49F cells. The expression of Ptprj remained unchanged under different concentrations of GJ103 (Figure 7H,I).

GJ103 sodium is a read‐through compound that can induce read through of premature stop codons and also has potential for the research of genetic disorders caused by nonsense mutations.^[^ ^24^ ^]^ The role of GJ103 in kidney disease has not been reported. We found that different concentrations of GJ103 had little effect on cell viability (Figure 7J). Subsequently, the cytoprotective effect of GJ103 was evaluated. NRK49F cells were pretreated with 5, 10, 20, 40 µm GJ103 for 2 h, respectively, and then stimulated with TGF‐β1. We found that the phosphorylation level of PDGFRβ was significantly inhibited at GJ103 concentrations above 20 µm (Figure 7K,L). Along with the decrease of p‐PDGFRβ expression, protein levels of fibrosis markers FN1 were repressed (Figure 7M,N).

**Figure 8:**
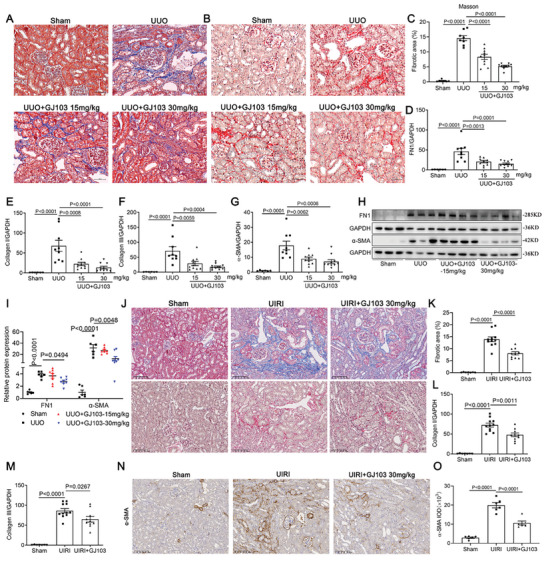
Ptprj activator protected against renal fibrosis in UUO and UIRI models. A,B) Photomicrographs illustrating Masson trichrome staining (blue) and Picrosirius red staining of renal tissue (×200). Scale bar, 50 µm. C) Quantitative data of fibrosis area according to Masson staining. D–G) qRT‐PCR analysis of renal FN1, Collagen I, Collagen III, α‐SMA mRNA levels (*n *= 6 in Sham group, *n *= 9 in UUO group, *n *= 10 in GJ103 treatment group). H,I) Western blotting analysis of FN1 and α‐SMA levels (*n *= 6 in Sham or UUO group, *n *= 8 in GJ103 treatment group). J) Representative images of Masson trichrome staining (upper) and Picrosirius red staining (down) of renal tissue (×200). Scale bar, 100 µm. K) Quantitative data of fibrosis area according to Masson staining (*n *= 7 in Sham group, *n *= 11 in UIRI group, *n *= 10 in UIRI+GJ103 group). L,M) qRT‐PCR analysis of renal Collagen I and Collagen III mRNA levels (*n *= 7 in Sham group, *n *= 11 in UIRI group, *n *= 10 in UIRI+GJ103 group). N,O) Representative immunohistochemical images of α‐SMA staining and quantitative analysis of α‐SMA (*n *= 6 in each group). Scale bar, 100 µm. Data are presented as mean ± SEM. Statistical analysis was performed using One‐way ANOVA followed by Bonferroni. The P‐values were shown in the figures. Abbreviations: UUO, unilateral ureteral obstruction; UIRI, unilateral ischemia/reperfusion injury.

We then validated the protective function of GJ103 in animal models. For intervention of UUO model, the mice were pretreated with GJ103 at 15 and 30 mg kg^−1^ day^−1^ before UUO surgery. Then the mice were treated daily for 7 consecutive days and sacrificed. Measurements of serum concentrations of BUN, Scr, aspartate aminotransferase (AST), alanine aminotransferase (ALT), creatine kinase‐MB (CK‐MB) and lactate dehydrogenase (LDH) indicated that the therapeutic dose of GJ103 did not cause significant renal, hepatic, cardiac, or systemic toxicity (Figure S6A–G, Supporting Information). Masson's trichrome and Picrosirus Red staining exhibited that GJ103 treatment, especially high concentration treatment, significantly attenuated the degree of renal interstitial fibrosis (**Figure** **8**A–C). The deposition of extracellular matrix components FN1, Collagen I, Collagen III and α‐SMA as measured at both the mRNA level (Figure 8D–G) and protein level (Figure 8H,I) also decreased after GJ103 treatment, especially in the high dose group. We also examined the effect of GJ103 in the UIRI model, and consistent with the results obtained in the UUO model, GJ103 (30 mg kg^−1^ day^−1^) also improved UIRI‐induced renal fibrosis, manifested in improved Masson's trichrome and Picrosirus Red staining (Figure 8J,K), reduced extracellular matrix deposition (Figure 8L,M), and alleviated α‐SMA expression (Figure 8N,O). Therapeutic doses of GJ103 also did not show significant renal, hepatic, cardiac, or systemic toxicity in UIRI model (Figure S6H–M, Supporting Information).

### 2.7 Tubular‐Specific Overexpression of Meis1 Attenuated Renal Fibrosis in UUO Model, While Inhibition of Meis1 did the Opposite

It is acknowledged that renal fibrosis can also be indirectly caused by the injury of renal tubular epithelial cells, and the expression of Meis1 in renal tubules was also increased. To study the role of Meis1 in renal tubules, we generated mice strain with tubular‐specific overexpression of Meis1 and established UUO model. We found specific overexpression of Meis1 in tubular cells (Figure S7A, Supporting Information) attenuated the degree of renal interstitial fibrosis and inflammation, as determined by Masson's trichrome (Figure S7B,C, Supporting Information) and the expression levels of FN1, α‐SMA, and TNF‐α (Figure S7D–H, Supporting Information).

Considering the antifibrotic effect of Meis1 in fibroblasts and renal tubules, we used MEISi‐2, a reported specific inhibitor of Meis1,^[^ ^25^ ^]^ for intraperitoneal injection from day 1 to day7 after UUO surgery (0.4 mg kg^−1^ d^−1^). We found MEISi‐2 aggravated UUO‐induced renal fibrosis (Figure S8A–E, Supporting Information). We then confirmed the function of MEISi‐2 in NRK‐49F cells and found that the antifibrotic protective effect of Meis1 against TGF‐β1 stimulation disappeared after MEISi‐2 treatment (Figure S8F–H, Supporting Information). These results suggest that Meis1 plays an antifibrotic role in both fibroblasts and tubular cells.

## 3 Discussion

**Figure 9:**
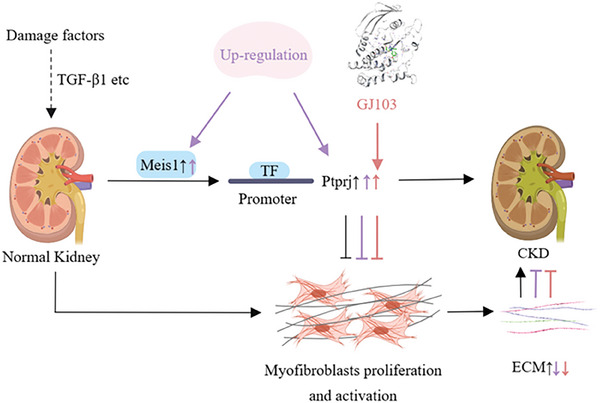
Schematic diagram illustrating the antifibrotic role of the Meis1/Ptprj pathway in kidney. In the context of chronic kidney disease (CKD), various pro‐fibrotic factors, including TGF‐β1, play a role in activating fibroblasts and promoting renal fibrosis through a series of pathological mechanisms. Additionally, these factors also activate the Meis1/Ptprj pathway in a compensatory manner to counteract the excessive proliferation and activation of fibroblasts. However, the endogenous activation of this pathway is insufficient to effectively counteract the occurrence and progression of renal fibrosis. Therefore, the active up‐regulation or activation of Meis1/Ptprj through exogenous pathways can significantly inhibit the proliferation and activation of fibroblasts. Created with MedPeer (www.medpeer.cn).

Upon injury, maladaptive TECs contribute to alterations in the microenvironment of the tubulointerstitial space in which myofibroblasts are activated and produce substantial amounts of ECM.^[^ ^26^ ^]^ On the other hand, activated myofibroblasts can also exert profibrotic effects on tubular cells. The accumulation of ECM leads to biomechanical changes of the interstitium, including increased matrix rigidity, which in turn causes augmented TGF‐β1 expression and partial epithelial‐mesenchymal transition (EMT) behavior.^[^ ^27^ ^]^ In addition, resident fibroblasts transform at the expense of erythropoietin production into myofibroblasts in response to injury and produce numerous ECM components.^[^ ^28^ ^]^ The ECM can increase the diffusion distance of oxygen, thereby aggravating hypoxia in TECs.^[^ ^28^ ^]^ Therefore, inhibiting the proliferation and activation of myofibroblasts is a top priority for controlling and improving renal fibrosis. In the current study, we confirmed the up‐regulation of Meis1 expression in renal fibroblasts clearly delayed the progression of renal fibrosis and did so by regulating Ptprj to inhibit fibroblast proliferation and activation, which retarded the malignant development of CKD. The diagram in **Figure** **9** illustrates the function of Meis1 in the kidney.

Studies have shown that Meis1 expression in the interstitium is essential during nephrogenesis, but its function in the kidney remains unclear. Chang‐Panesso et al. showed that Meis1 expression occurs mainly in perivascular fibroblasts and pericytes of mice and both acute and chronic injury can specifically up‐regulated Meis1 expression.^[^ ^29^ ^]^ Moreover, they also found that Meis1 is primarily expressed in the cell nucleus and only minimally expressed in the cytoplasm. Their results are not completely consistent with our current findings. For instance, we also found that Meis1 was mainly expressed in the nucleus; however, it was not difficult for us to detect Meis1 expression in the cytoplasm of both human renal biopsy specimens and mouse kidneys. Surprisingly, we also detected Meis1 expression in tubules as well as in the renal interstitium. Both our current studies and those of Chang‐Panesso et al.^[^ ^29^ ^]^ consistently demonstrated that the expression level of Meis1 increased in injured kidneys; however, there are differences regarding the location of the Meis1. Our human kidney biopsy specimens were obtained from the kidney tissues of children aged 1–15 years with CKD that was caused by different aetiologies and the mice we used all were in a C57BL/6J background. We observed the same results in adult CKD kidney biopsy specimens, most of which were diagnosed with IgAN. Meanwhile, the human kidney specimen used by Chang‐Panesso and colleagues came from a 62‐yr‐old man with an Scr level of 3.3 mg dL^−1^ at time of collection and their experimental mice included a mixed background. We speculate that differences in the inclusion criteria of the samples for the two studies are probably an important factor for the inconsistent conclusions. Meis1 is an important transcription factor for kidney development and its level and location of expression may change with age. Furthermore, we believe that not only does the expression level of Meis1 change under different injury conditions, but Meis1 may function through different levels of nuclear translocation. This may also explain our observation of Meis1 being expressed in the cytoplasm. Obviously, whether the varying localization of Meis1 is related to its nuclear translocation remains to be further explored in the future.

Our findings suggested that Meis1 could transcriptionally regulate Ptprj to alleviate renal fibrosis. Ptprj is widely expressed in haematopoietic cells, endothelial cells, fibroblasts, thyroid cells, and other cell types^[^ ^30^ ^]^ and it exhibits powerful tumor suppressive activity in multiple tumors. Takahashi et al. generated an agonistic anti‐Ptprj antibody that can clearly prevent a decrease in the number of podocytes, inhibit nephrin expression, reduced the level of FN1 in the glomerulus, and reduce renal macrophage infiltration by down‐regulating epidermal growth factor receptor (EGFR) signals in podocytes. This results in diabetic nephropathy improving in mice treated with the antibody.^[^ ^31^ ^]^ This is consistent with the phenomenon we observed when Ptprj heterozygous knockout aggravated renal fibrosis in UUO mice. These results further confirm that Meis1 control of Ptprj can produce a unique protective effect in the kidney. Previous reports have shown that Ptprj can dephosphorylate various receptor tyrosine kinases to inhibit multiple mitogenic signals, thereby exhibiting significant anti‐proliferative functions.^[^ ^32^ ^]^ In the present study, total protein expression and phosphorylation levels of PDGFRβ were both clearly inhibited by Ptprj. Therefore, we suggest that Ptprj may regulate the proliferative state of fibroblasts by interfering with the activity of PDGFRβ. However, we cannot exclude the possibility that Ptprj is involved in renal fibrosis through other downstream signaling pathways. For example, Ptprj is known to exert an anti‐fibrotic effect in pulmonary fibrosis through interactions with syndecan‐2.^[^ ^33^ ^]^ Furthermore, Ptprj can also regulate the activity of the nonreceptor tyrosine kinase protein proto‐oncogene tyrosine protein kinase Src^[^ ^34^ ^]^ and numerous studies have shown that activated Src is a pivotal molecule in multiple fibrosis pathways.^[^ ^35^ ^]^ Therefore, it is not difficult to see that Ptprj may have a long‐ignored, but vital role in organ fibrosis.

Given the acknowledged protective role of PTPRJ and the absence of specific PTPRJ activators in previous studies, our investigation involved the implementation of molecular docking techniques to identify potential PTPRJ activators as our previous reported.^[^ ^36^ ^]^ Subsequently, we conducted substrate protease activity assays, in vitro dephosphorylation of the PDGFRβ, and SPR assays to assess the activation potential of high‐scoring candidate molecules on PTPRJ. Among the identified candidates, the novel compound GJ103 exhibited the most promising PTPRJ activation and displayed a discernible interaction with PTPRJ. Notably, GJ103 demonstrated a significant antifibrotic effect on fibroblasts in vitro, which is highly encouraging. In an in vivo setting, GJ103 has been shown to antagonize renal fibrosis in mice with UUO and UIRI. However, the specific mechanism by which GJ103 activates PTPRJ remains unclear. It has been observed that there was little change in PTPRJ protein level after GJ103 stimulation, which suggests that GJ103 may only activate the enzyme activity of PTPRJ. In addition, the tyrosine protein phosphatase domain of PTPRJ spans amino acid residues 1041–1298,^[^ ^37^ ^]^ GJ103 may activate downstream PTPRJ signal by affecting the conformation of PTPRJ protein, as has been reported for the activator of PI3Kα.^[^ ^38^ ^]^

Although the underlying mechanism of renal fibrosis is not fully understood, the proliferation and activation of renal interstitial myofibroblasts and the excessive deposition of ECM are considered to be main pathological events in the progression of renal fibrosis. By employing bioinformatics, improved biochemical methods, and advanced genetic engineering tools, our research group has demonstrated that Meis1 was able to attenuate renal fibrosis by regulating Ptprj/PDGFRβ pathway to inhibit fibroblast proliferation and activation. This suggests Meis1/Ptprj/PDGFRβ pathway may be a potential new therapeutic target for retarding CKD. It has been reported that elevated levels of soluble PDGFRβ (sPDGFRβ) in cerebrospinal fluid (CSF) are an early biomarker of human Alzheimer's disease.^[^ ^39^ ^]^ Therefore, detecting soluble PDGFRβ in blood or urine could has a potential as biomarker for the diagnosis of CKD in the future.

The limitation of our study is that whether the effect in our study of Meis 1 regulating Ptprj to improve renal fibrosis depended solely on its inhibitory effects on mitogenic signals such as PDGFRβ remains to be determined. It is possible that Ptprj is able to act as a central signaling molecule by regulating multiple downstream pathways to form a signaling network in populations of activated fibroblast and other cell types. In addition, how Ptprj small molecule activators specifically activate Ptprj remains to be explored further in the future studies.

## 4 Experimental Section

### Reagents and Antibodies

Dulbecco's modified Eagle's medium (DMEM, Cat No. C11995500BT), fetal bovine serum (FBS, Cat No. 10 091 148), and 0.25% trypsin‐0.02% EDTA (Cat No. 25200‐056) were purchased from GIBCO (Waltham, MA, USA). Antibodies against FN1 (Cat No. ab2413), α‐SMA (Cat No. ab7817), c‐Myc (Cat No. ab32072), PAI1 (Cat No. ab222754), and Meis1 (Cat No. ab19867) were purchased from Abcam (Cambridge, MA, USA). Antibodies used to detect Meis1 expression in adult CKD, as well as in cytoplasm and nucleus by western blot assay were purchased from Origene (Cat No. TA809619S). Anti‐PDGFRβ (Cat No. A19531) and Vimentin (Cat No. A19607) antibodies were obtained from Abclonal (Wuhan, China). Anti‐FSP1 (Cat No. 66489‐1‐lg) was from Proteintech Group (Rosemont, IL, USA). Anti‐collagen III (Cat No. bs‐0549R), Collagen I (Cat No. bs‐10423R), Ptprj (Cat No. bs‐2567R), and GAPDH (Cat No. bs‐0755R) were purchased from Bioss (Beijing, China). Antibody against CyclinD1 (Cat No. 2978S), Anti‐p‐PDGFRβ (Tyr1021) (Cat No. 2227S), SimpleChIP Enzymatic Chromatin IP Kit (Magnetic Beads, Cat No. 9003S), and SimpleChIP Universal qPCR Master Mix (Cat No. 88989P) were from Cell Signaling Technology (Boston, MA, USA). TGF‐β1 was from Pepro Tech (Cat No. 100‐21‐10 µg, Rocky Hill, USA). Folic acid (Cat No. F8758) and anti‐flag (Cat No. F1804) antibody were bought from Sigma‐Aldrich (St. Louis, MO, USA). Picrosirius Red staining Kit was from BestBio (Shanghai, China). Alexa Fluor Plus 555 phalloidin (Cat No. A30106) was from ThermoFisher (Waltham, MA, USA). BeyoClick EdU‐488 cell proliferation detection kit (Cat No. C0071S) was from Beyotime (Shanghai, China). CCK‐8 Cell Counting Kit (Cat No. A311‐01) was from Vazyme (Nanjing, China). GJ103 (Cat No. HY‐101203A) and Lamin B1 antibody (Cat No. HY‐P80205) were bought from MedChemExpress (Monmouth Junction, NJ, USA). Phalloidin iFluor 555 (Cat No. 40737ES75) was bought from Yeasen Biotechnology (Shanghai, China).

### Ethical Statement

The protocol for the use of biopsy samples and nephrectomy tissue from children with CKD was approved by the Human Subjects Committee of Children's Hospital of the Nanjing Medical University (the ethical approval number: 202206126‐1). The use of biopsy samples from adult patients with CKD was approved by the Human Subjects Committee of the First Affiliated Hospital of Nanjing Medical University (the ethical approval number: 2021‐SR‐398). All patients provided written informed consent. All animal procedures were approved by the Institutional Animal Care and Use Committee at Nanjing Medical University, China (the ethical approval number: IACUC‐2102005, 2102005‐3).

### Patients

Renal biopsy samples were obtained from CKD patients who were undergoing diagnostic evaluation at the Department of Nephrology, Children's Hospital of Nanjing Medical University or the First Affiliated Hospital of Nanjing Medical University. The inclusion criterion was presence of at least ten glomeruli in the paraffin‐embedded tissue sample available for histological sectioning. A total of 26 subjects (17 children, 9 adults) were enrolled; the pathological diagnosis was IgA nephropathy (IgAN), Lupus nephritis (LN), ANCA‐associated vasculitis (AAV), Henoch‐Schonlein purpura nephritis (HSPN), Tubulointerstitial nephropathy, sclerosing glomerulonephritis, focal segmental glomerulosclerosis (FSGS), podocytopathy or diabetic nephropathy (DN). The patient information is listed in Table S1 (Supporting Information). Nontumor renal tissue from patients who had renal tumors and underwent nephrectomy were used as normal controls.

### Animals

The mice were housed in a temperature‐controlled room (19–21 °C) and subjected to a 12 h light‐dark cycle. They were provided with a standard rodent diet and unrestricted access to drinking water. All animal procedures were approved by the Institutional Animal Care and Use Committee at Nanjing Medical University, China.

### Generation of a Fibroblast Meis1 Conditional Knockin Mouse (cKI) Strain

Generation of a fibroblast Meis1 Conditional Knockin Mouse (cKI) Strain. The Meis1flox/flox knock‐in mice were purchased from GemPharmatech. Using the CRISPR system, insert the transcription termination signal original STOP between the CAG promoter and Meis1 sequence, construct the CAG‐loxp‐STOP‐loxp‐Meis1 skeleton vector and transfer it into the male pronucleus of the fertilized egg to construct the transgenic mice, and then crossed with the S100A4‐Cre transgenic mice (J004128, purchased from the Jackson Laboratory) to generate F1 (Meis1flox/+; S100A4‐Cre+). Then, F1 mated backcross with the Meis1flox/flox knock‐in mice to generate fibroblast Meis1 conditional knock‐in (Meis1flox/flox; S100A4‐Cre+, cKI) mice. The wide type (Meis1flox/flox; S100A4‐Cre‐, WT) from the same litters were used as controls.

### Generation of Ptprj^+/−^ Mouse Strain

Ptprj‐KO mice (Strain NO. T035101, genetic background: C57BL/6J) were purchased from GemPharmatech (Nanjing, China). Heterozygous F0 generation mice were obtained by freezing sperm resuscitation. After hybridization of F0 generation mice with WT mice (C57BL/6J), a sufficient number of F1 generation heterozygous mice (Ptprj^+/−^) were obtained for experiment (8 weeks old, male and female).

### Unilateral Ureteral Obstruction (UUO) Model

As previously reported,^[^ ^36^ ^]^ following administration of anesthesia, the mice were immobilized on the surgical plate in a supine position. The abdominal surgical site was sterilized as per standard procedure, a midabdominal incision was performed, sequentially cutting through the skin and muscle layers to expose the kidney and ureter. The left ureter was ligated near ureteropelvic junction with a 4‐0 silk suture, followed by routine suturing of the muscle and skin layers. Mice were sacrificed after 7 days.

### Unilateral Ischemia/Reperfusion Injury (UIRI) Model

As previously reported,^[^ ^36^ ^]^ Meis1‐cKI or WT male mice were anesthetized, and the left kidney was exposed through a median ventral incision. Ischemia was induced by clamping the renal pedicle with nontraumatic microaneurysm clamps. After a duration of 32 min, the artery clamp was removed, allowing for restoration of blood flow and observation of kidney recovery. During the experiment, body temperature of mice was controlled at 36.5–37.5 °C. Mice were sacrificed after 3 weeks.

### Folic Acid (FA) Model

In this experimental model, kidney fibrosis was induced through intraperitoneal injection of FA (250 mg kg^−1^) dissolved in sodium bicarbonate (3 × 10^−1^ m NaHCO3) as previously described.^[^ ^40^ ^]^ Control mice were intraperitoneally injected with the same amount of sodium bicarbonate (3 × 10^−1^ m NaHCO3, vehicle). Mice were sacrificed after 4 weeks.

### Pharmaceutical Treatment

Wild type C57BL/6J mice (8 weeks old, male) were purchased from GemPharmatech (Nanjing, China). For intervention of UUO model, the mice were pretreated with GJ103 at 15 and 30 mg kg^−1^ day^−1^ via intraperitoneal (i.p.) injection 24 h before UUO surgery. Then the mice were treated daily for 7 consecutive days and sacrificed. For intervention of UIRI, the mice were treated with GJ103 30 mg kg^−1^ day^−1^ via i.p. injection 24 h before UIRI surgery. The mice were treated daily for 14 consecutive days and sacrificed.

### Cell Culture Studies

NRK‐49F cells were grown in DMEM medium supplemented with 10% FBS and maintained at 37 °C with 5% CO_2_ in a humidified incubator. The cells were subcultured at 70–80% confluence using 0.25% trypsin‐0.02% EDTA. In certain experiments, cells were transfected with Meis1, shMeis1 or Ptprj plasmids using lipo3000 (Cat No. L3000015, ThermoFisher, IL, USA) and treated with recombinant human TGF‐β1 (10 ng mL^−1^) after 6–8 h. Cells were collected 24 h later for mRNA analysis or 48 h later for protein analysis.

### Immunohistochemistry

Kidney sections (3 µm thick) were mounted on slides. The slides were deparaffinized and rehydrated using xylene and graded ethanol series using the standard method. The sections were incubated in 3% hydrogen peroxide for 20 min, boiled in modified Citrate Antigen Retrieval Solution (Beyotime, Cat No. P0086, Beijing, China) for 1–2 min as previously reported.^[^ ^41^ ^]^ The slides were then incubated overnight at 4 °C with primary antibodies against Meis1 (1:400), α‐SMA (1:2000), and Ptprj (1:250). The biotin‐conjugated secondary antibody was applied after washing with phosphate buffer saline. The chromogenic reaction was carried out with diaminobenzidine (DAB), and the nuclei were counterstained with hematoxylin. Image ProPlus was used for image analysis and quantification.

### Immunofluorescence Staining

Kidney tissues on slides were processed in accordance with immunohistochemistry. After permeabilized with 0.1% Triton X‐100, the primary antibody Meis1 (1:1000) and FSP1 (1:250) were added to the slides and incubated overnight at 4 °C; the secondary antibody goat Anti‐rabbit IgG H&L (Alexa Fluor 555) and goat Anti‐mouse IgG H&L (Alexa Fluor 488) were applied after washing with PBST. Nuclei were stained with DAPI. After being washed, the slides were viewed under a laser scanning confocal microscope (Zeiss), photographed and recorded. The co‐staining procedure of Ptprj (1:250) and FSP1 was the same as above.

NRK‐49F were cultured on glass‐bottom cell culture dishes (NEST, China) following the specific experimental treatment. Then, after several washes with PBS, the cells were fixed with 4% paraformaldehyde for 15 min at room temperature, extensively washed with PBS, permeabilized with PBS + 1% Triton X‐100 for 10 min and blocked with 2% BSA for 1 h. The primary antibody α‐SMA (Cell Signaling Technology, Cat No. 19245S) was added to the dish at a 1:300 dilution and incubated in a humid chamber overnight at 4 °C; the secondary antibody and phalloidin iFluor 555 (1:1000) were applied after washing with PBST. Then, the cells were viewed under a laser scanning confocal microscope (Zeiss), photographed and recorded.

### Nuclear and Cytoplasmic Extraction

The nuclear and cytoplasmic extraction was prepared using an NE‐PER Nuclear and Cytoplasmic Extraction Reagents (Cat No. 78 833, ThermoFisher) according to the manufacturer's instruction. Briefly, the 30 mg of fresh tissue was cut into small pieces, washed with PBS and centrifuged at 500 g for 5 min. Homogenize tissue using a Dounce homogenizer in 400 µl CER I. The suspension was incubated on ice for 10 min followed by the addition of 22 µl CER II, vortexed on the highest setting for 5 s, incubated on ice for 1 min and centrifuged for 5 min at 16 000 g. The supernatant fraction was cytoplasmic extract and transferred them to a pre‐chilled tube. Suspend the insoluble (pellet) fraction, which contains nuclei, in ice‐cold NER and place the sample on ice and continue vortexing for 15 s every 10 min, for a total of 40 min, then centrifuged for 10 min at 16 000 g. The resulting supernatant, constituting the nuclear extract, was used for the subsequent experiments.

### Kidney Histopathological Analysis

Kidney tissues were fixed overnight in 4% paraformaldehyde (PFA), dehydrated, embedded in paraffin, and cut into 3 µm sections transversely. After deparaffinisation, the kidney sections were stained with Masson trichrome and Picrosirius red staining as previously reported.^[^ ^41^ ^]^

### Quantitative Real‐Time PCR

According to the manufacturer's protocol, RNA isoPlus reagent (Cat No. 9109, TaKaRa Biotechnology, Japan) was used to extract total RNA from tissues or cells. cDNA was reverse transcribed with HiScript II Q RT SuperMix for qPCR (Cat No. R222‐01, Vazyme Biotechnology, Nanjing, China). Real‐time PCR amplification was carried out through the ABI 7500 real‐time PCR detection system using AceQ qPCR SYBR Green Master Mix (Cat No. Q131‐02, Vazyme Biotechnology). The temperature cycling conditions were 95 °C for 10 min followed by 40 cycles of 95 °C for 15 s and 60 °C for 1 min. The relative expression level of mRNA was normalized to the relative expression of GAPDH and calculated using the 2 ^−ΔΔCt^ method as previously reported.^[^ ^41^ ^]^ The primers were designed by Primer 5.0 software (http://Frodo.wi.mit.edu) and the primer sequences are shown in Table S2 (Supporting Information).

### Western Blotting

Homogenize tissues or cell lysates with RIPA buffer containing 1×protease inhibitor (Cat No. 0 469 313 2001, Roche, Basel, Switzerland) and 1×phosphatase inhibitor (Cat No. 0 490 683 7001, Roche). The samples were then centrifuged at 5000 rpm for 30 min. BCA protein assay kit (Cat No. 23 227, ThermoFisher) was used to determine the protein concentration. Equivalent samples were used for primary antibodies against Meis1 (1:1000), FN1 (1:1000), Collagen I (1:1000), Collagen III (1:1000), PDGFRβ (1:1000), p‐PDGFRβ (1:1000), PAI1 (1:1000), c‐Myc (1:1000), Ptprj (1:500), Vimentin (1:10 000), Lamin B1 (1:1000), α‐SMA (1:3000), and GAPDH (1:2000), and then added HRP‐labeled secondary antibody (1:5000). The immunoreactive bands were visualized using the Amersham Biosciences ECL detection system. Densitometric analysis was conducted through the quantification of band intensity, followed by normalization to the corresponding GAPDH band using Image Lab software.

### EdU Staining

NRK‐49F were cultured on 6‐well plate with EdU working solution (1 × 10^−5^ m) and incubated for 2 h. Remove the culture solution, add 1 ml fixing solution, and fix at room temperature for 15 min. Then add 1 ml of permeable solution and incubate at room temperature for 10 min. Finally add 0.5 ml Click reaction solution per well and incubate at room temperature for 30 min away from light. Nuclei were stained with DAPI.

### Luciferase Reporter Assay

The Meis1 or shMeis1 or the control plasmids were transfected into NRK‐49F cells, followed by transfection with the pGL4.19‐Ptprj promoter and pRL‐TK plasmids (Public Protein/Plasmid Library, China). Luciferase assays were performed using the reporter assay system (Cat No. E1910, Promega, Madison, WI, USA) according to the manufacturer's protocol: after removing the medium, cells were washed and lysed before being analyzed in a GloMax 96 Microplate Luminometer with Dual Injector System (Promega). Firefly luciferase was measured first with Luciferase Assay Reagent II, followed by quenching the reaction and initiating the Renilla luciferase reaction with Stop & Glo Reagent. Relative luciferase activity was calculated as the ratio of firefly to Renilla luciferase activity (F/R).

### ChIP Assay

Cross‐linked chromatin was prepared from 293T cells and performed using the SimpleChIP Enzymatic Chromatin IP Kit (Magnetic Beads) according to the manufacturer's protocol. In brief, ChIP was performed using 20 µg of cross‐linked chromatin (sheared by sonication and micrococcal nuclease to 300‐450‐bp fragments) per reaction and 2 µg of antibody to Meis1 or positive control Histone H3 or negative rabbit IgG control for immunoprecipitation. A −2000∼+100‐bp sequence upstream of Ptprj transcription start site was analyzed using JASPAR (http://jaspar.genereg.net/) for potential transcription‐factor‐binding sites. ChIP primers were then designed to amplify Ptprj promoter regions and the primers listed in Table S3 (Supporting Information).

### Molecular Docking

The tyrosine protein phosphatase domain of PTPRJ spans amino acid residues 1041–1298. The X‐ray structure of the catalytic domain of human PTPRJ has been determined, with PDB IDs 2CFV and 2NZ6.^[^ ^42^ ^]^ The structure represented by 2NZ6 is of the wild‐type, lacking any small molecules at the binding site. In contrast, the active site of 2CFV has undergone a mutation, with Cys1298 being replaced by Ser, and the binding site being occupied by phosphate. This allows for the fixation and resolution of the surrounding flexible loop region. Upon aligning the two structures, it was observed that the overall framework remains largely unchanged, with no alterations in the side chain positions of the wild‐type Cys and mutated Ser residues. Therefore, Ser1298 in 2CFV was mutated back to Cys and locally optimized. The optimized structure was used as a receptor for subsequent virtual screening of MCE‐new Bioactive Compound Library and MCE online Bioactive Compound Library with 9372 compounds in total. After LigPrep preparation, 9322 and 11 510 structures (different protonation states and tautomers) were selected for docking at the SP precision by Glide. This work was supported by Center for Excellence in Molecular Cell Science (Shanghai, China).

### In Vitro Dephosphorylation of the PDGFRβ

NRK49F cells were stimulated with TGF‐β1(10 ng mL^−1^) at fusion to 70%. Twenty four hours after stimulation, cells were washed twice with ice‐cold PBS. Cells were lysed with IP lysis buffer (Thermo, Cat No. 87 787) supplemented with protease inhibitor (Roche, Cat No. 0 469 313 2001) and phosphatase inhibitor (Roche, Cat No. 0 490 683 7001) cocktail on ice. The lysates were clarified by centrifugation and then mixed with PDGFRβ antibody (CST, Cat No. 3169) and magnetic beads (MCE, Cat No. HY‐K0202) overnight at 4 °C. Immunocomplexes were collected on beads, rinsed four times with washing buffer. Aliquots of immunosorbent PDGFRβ immobilized on magnetic beads were suspended in 40 µl of buffer containing 2 × 10^−2^ m imidazole, pH 7.4, 0.05% Triton X‐100, 1 × 10^−2^ m DTT, 0.1 mg mL^−1^ bovine serum albumin, and incubated with purified protein DEP1‐His (MCE, Cat No. HY‐P74200), GJ103 (MCE, Cat No. HY‐101203), and AS252424 (MCE, Cat No. HY‐13532) for 30 min at 30 °C. Total reaction volume was 60 µl. Dephosphorylation was terminated by the addition of SDS Loading Buffer and analyzed by western blotting. This experimental process was based on the literature report^[^ ^15^ ^]^ and modified slightly.

### Surface Plasmon Resonance (SPR)

*Coupling of the Target Protein PTPRJ*: The binding affinity of GJ103 with PTPRJ were determined using the BIAcore T200 SPR biosensor systems with a NTA Sensor Chip (Cytiva). In order to avoid the inactivation of the target protein during the amino coupling process, NTA chip was used in this experiment. NiCl_2_ (5 × 10^−3^ m) was passed through the surface of the test channel of the chip with a flow rate of 30 µl min^−1^ and injection time of 120 s. After capturing nickel ions in the test channel, the target protein PTPRJ was diluted to 20 µg mL^−1^ with analytical buffer PBS at a flow rate of 10 µl min^−1^ and a duration of 240 s, and PTPRJ protein was captured to an appropriate level.

*Conditions of Small Molecule Compound GJ103*: The binding properties of target protein PTPRJ and small molecule compound GJ103 were preliminatively determined and evaluated by manual mode. 1 × 10^−4^ m was determined as the maximum analytical concentration of GJ103, and 7 analytical concentrations were set according to double gradient dilution. The concentration gradients were 0, 3.125 × 10 ^−6^ , 6.25 × 10 ^−6^ , 1.25 × 10 ^−5^ , 2.5 × 10 ^−5^ , 5 × 10 ^−5^ , and 1 × 10^−4^ m, respectively. The flow rate was set at 30 µl min^−1^. The binding time was 120 s and the dissociation time was 120 s.

*Dynamic Parameter Determination*: The experiment was run in multiple cycles, and the obtained data were fitted by BIAcore T200 analysis software. The fitting model adopted was 1:1 Langmuir combined model to determine its dynamic constants. This work was supported by Wayen Biotechnologies Inc (Shanghai, China).

### Statistical Analysis

Data are expressed as mean ± SEM. Quantifications of immunoblotting were performed by ImageJ. Statistical analyses were performed with GraphPad Prism (version 8.0, GraphPad Software, SanDiego, CA). A two‐tailed Student's t‐test was used to compare differences between two groups. For comparisons among multiple groups, one‐way ANOVA followed by Bonferroni was applied. *p* < 0.05 was considered statistically significant. All statistical details regarding P‐value were shown in the figures and n can be found in the figure legends.

## Conflict of Interest

The authors declare no conflict of interest.

## Author Contributions

M.B. and S.X. contributed equally to this work. M.B. performed the experiments and wrote the manuscript. S.X., M.Z.J., Y.X.G., D.D.H., J.H., T.W., Y.G., and Y.Z. performed the experiments. Y.Z. helped interpret the data. S.M.H., Z.J.J., and A.H.Z. designed the experiment and interpreted the data. Z.J.J. and A.H.Z. revised the manuscript, and all authors read and approved the final manuscript.

## Supporting information

Supporting Information

## Data Availability

The data that support the findings of this study are available from the corresponding author upon reasonable request.
