# Supplementary material for: Meis1 Targets Protein Tyrosine Phosphatase Receptor J in Fibroblast to Retard Chronic Kidney Disease Progression
Source: Adv Sci (Weinh). 2024 Aug 20;11(39):2309754. doi: 10.1002/advs.202309754 (PMC11497016; doi:10.1002/advs.202309754)
Supplement: Supplementary file 1 — Supporting Information [file ADVS-11-2309754-s001.docx]

Supporting Information

**Meis1 Targets Protein Tyrosine Phosphatase Receptor J in Fibroblast to Retard Chronic Kidney Disease Progression**

*Mi Bai^#^, Shuang Xu^#^, Mingzhu Jiang, Yuxian Guo, Dandan Hu, Jia He, Ting Wang, Yu Zhang, Yan Guo, Yue Zhang, Songming Huang, Zhanjun Jia****^*^****, Aihua Zhang****^*^***

Experimental Section

Supplemental FigureS1-S8

Table S1-S3

**Experimental Section**

*Isolation and Cultivation of Primary Renal (myo)Fibroblasts:* Adult male WT and cKI mice (20-25 g) were employed to establish UUO animal model and were prepared for primary renal fibroblasts through a series of stainless-steel sieves (sieving method). After 7 days of UUO, mice were euthanized, and kidney tissues were harvested under aseptic conditions, washed with normal saline, minced into small pieces (1-3 mm) and gently ground on a 150-μm sieve. The filtrate was diluted with normal saline and then repeatedly passed through the 100-μm filter to collect interstitial fragments rich in renal tubules on the 100-μm sieve. The remaining tissue pieces were then resuspended in 5 ml DMEM with 20% FBS, 100 U mL^-1^ penicillin, 100 μg mL^-1^ streptomycin and 2 μg mL^-1^ α-fibroblast growth factor (α-FGF) (novoprotein, C043, Shanghai, China), placed into the gelatin-coated culture flasks and cultured in a 5% CO2 humidified incubator at 37°C. The cells subcultured at 60-80% confluence using 0.25% trypsin-0.02% EDTA. All experiments were performed on cells at passages 3–5. Cell purity was determined using FSP1 (1:250) immunofluorescence staining.

*High-throughput tail vein plasmid delivery*: Adult male C57BL/6J mice (20-25g) were provided by the Laboratory Animal Center of the Nanjing Medical University. As our previously reported,^[1]^ *Meis1* or *Ptprj* plasmids and the vehicle were dissolved in saline to a working concentration of 35μg mL^-1^ before injection. Then injected 2ml of the plasmids to the mice through the tail vein via high-throughput within 10 seconds. After 24 hours, the mice were conducted with UUO or UIRI models. The mice were housed in a temperature-controlled room (19–21°C) and subjected to a 12-hour light-dark cycle. They were provided with a standard rodent diet and unrestricted access to drinking water. All animal procedures were approved by the Institutional Animal Care and Use Committee at Nanjing Medical University, China.

*Generation of a tubular Meis1 conditional knockin mouse (Meis1-KAP cKI) strain:* The Meis1flox/flox knock-in mice were crossed with the KAP-Cre transgenic mice (#008781, purchased from the Jackson Laboratory) to generate F1 (Meis1flox/+; KAP-Cre+). Then, F1 mated backcross with the Meis1flox/flox knock-in mice to generate tubular Meis1 conditional knock-in (Meis1flox/flox; KAP-Cre+, Meis1-KAP cKI) mice. The wide type (Meis1flox/flox; KAP-Cre-, WT) from the same litters were used as controls. The mice were utilized in the generation of UUO model.

*MEISi-2 (Meis1 inhibitor) treatment:* In vivo: Wild type C57BL/6J mice (8 weeks old, male) were purchased from GemPharmatech (Nanjing, China). For intervention of UUO model, the mice were pretreated with MEISi-2 (0.4mg kg^-1^ day^-1^) via intraperitoneal (i.p.) injection 24h before UUO surgery. Then the mice were treated daily for 7 consecutive days and sacrificed. In vitro: NRK-49F cells were transfected with vehi or Meis1 plasmids using lipo3000 and treated with MEISi-2 (1μM) after 6–8h. Subsequently, TGF-β1 (10ng mL^-1^) was administered for 24h after 1 hour of treatment with MEISi-2.

References

[1] M. Wu, Q. Jin, X. Xu, J. Fan, W. Chen, M. Miao, R. Gu, S. Zhang, Y. Guo, S. Huang, Y. Zhang, A. Zhang, Z. Jia, *Adv. Sci. (Weinh)* **2023**, 10, e2301753.

**Supplemental Figures**


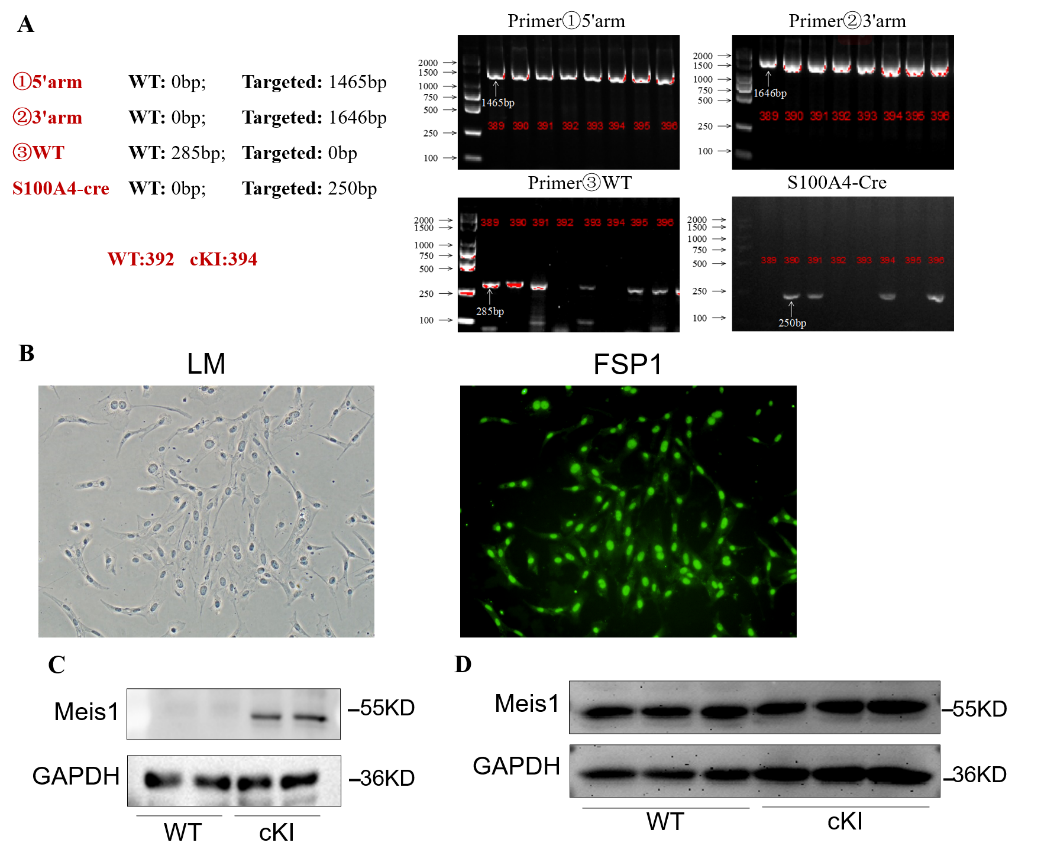


**Figure S1. Verification of Meis1 specific knockin mice.** (A) PCR analysis for genotyping the mice. WT (*Meis1*^flox/flox^; S100A4-Cre-): Primer① (+), Primer②(+), Primer③(-), S100A4-cre(-); cKI (*Meis1*^flox/flox^; S100A4-Cre+): Primer① (+), Primer②(+), Primer③(-), S100A4-cre(+). (B) Cell morphology and Fsp1 immunofluorescence staining confirmed the primary fibroblasts isolated from WT and cKI mice after UUO model. Scale bar, 50μm. (C) Western blotting analysis of Meis1 in primary fibroblasts isolated from WT and cKI mice after UUO model. (D) Proximal tubular cells were isolated from WT and cKI mice. Western blotting analysis showed Meis1 expression renal tubular cells had on difference between WT and cKI group (*n*=3). Abbreviations: LM, light microscope; Fsp1, fibroblast-specific protein 1.

**
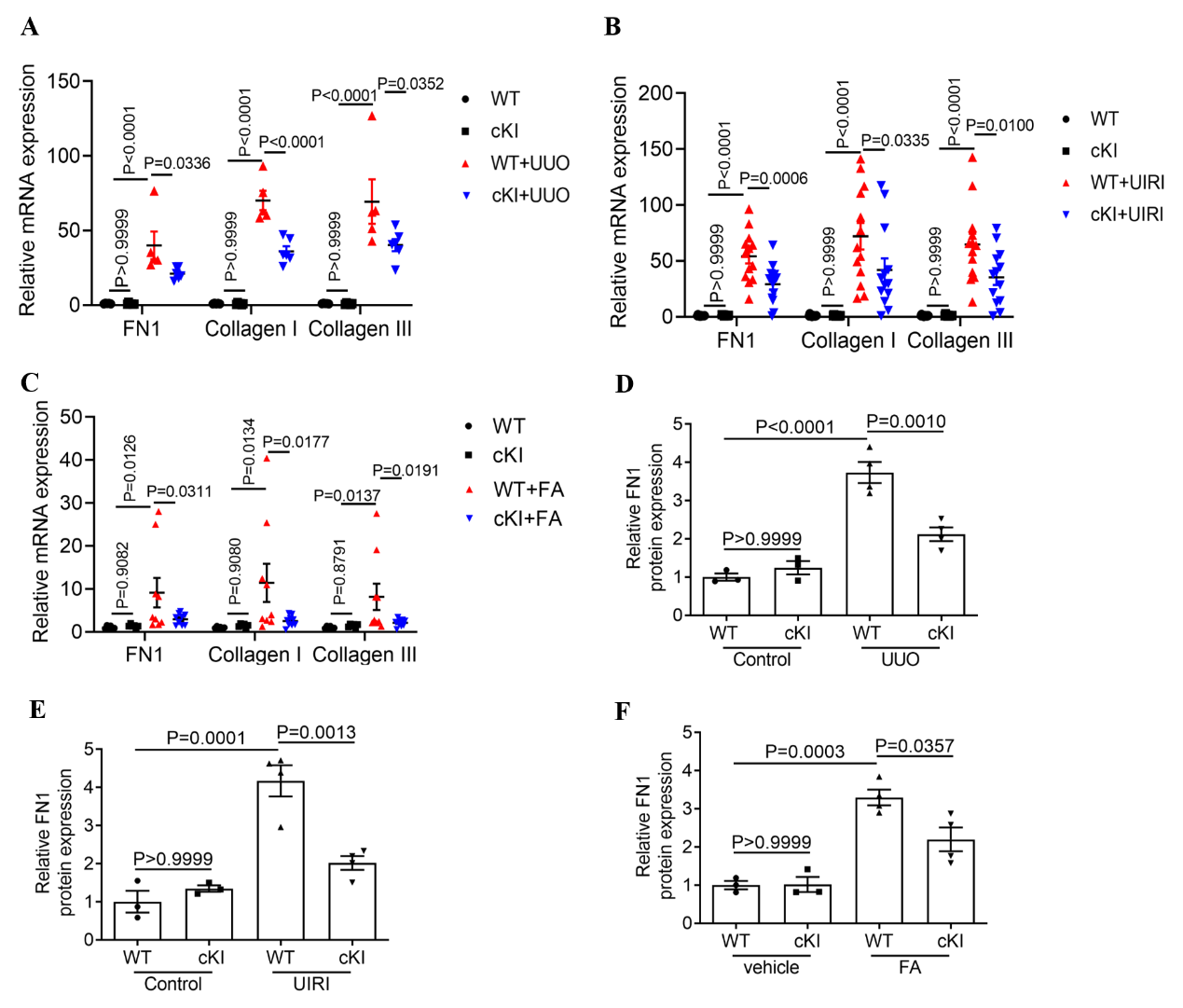
**

**Figure S2. Fibroblast-specific overexpression of Meis1 ameliorated renal fibrosis after CKD models.** (A) qRT-PCR analysis of renal FN1, Collagen I and Collagen III (*n*=5 in WT group, *n*=6 in cKI group) in WT and cKI mice after UUO model. (B) qRT-PCR analysis of renal FN1, Collagen I and Collagen III (*n*=11 in WT group, *n*=13 in cKI group) in WT and cKI mice after UIRI model. (C) mRNA expression of renal FN1, Collagen I and Collagen III in WT and cKI mice after FA model (*n*=6 in vehicle group, *n*=9 in FA group). (D-F) Quantitative data for western blotting analysis of FN1 in WT and cKI mice after UUO model (D), UIRI model (E) or FA model (F). N=3 in Control or vehicle group. N=4 in UUO, UIRI or FA group. Data are presented as mean ± SEM. Statistical analysis was performed using One-way ANOVA followed by Bonferroni. The P-values were shown in the figures. Abbreviations: UUO, unilateral ureteral obstruction; UIRI, unilateral ischemia/reperfusion injury; FA, folic acid.

**
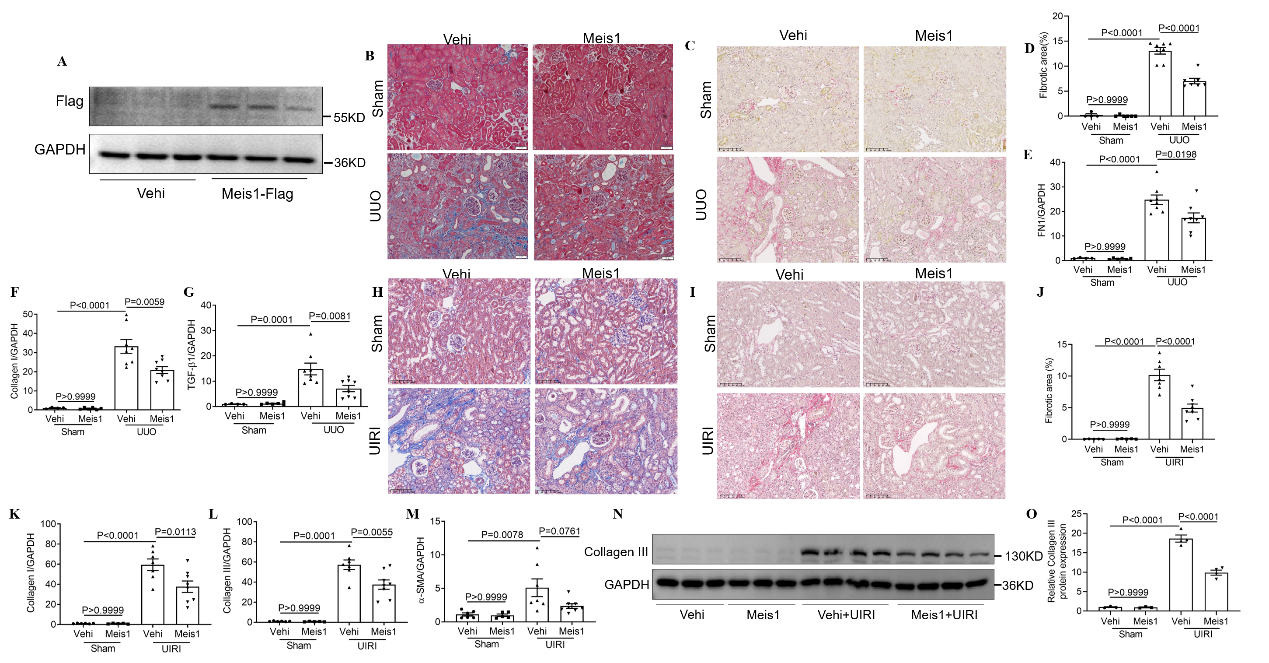
**

**Figure S3. Overexpression of Meis1 by high-throughput tail vein plasmid delivery in kidneys protected against UUO or UIRI-induced renal fibrosis.** (A) Western blotting analysis of Flag in kidney after tail vein injection of Vehi or Meis1-Flag plasmids (*n*=3). (B-C) Representative images of Masson trichrome staining and Sirius Red staining of renal tissue (×200) after UUO model. Scale bar, 50μm or 100μm. (D) Quantitative data of fibrosis area in UUO model (*n*=4 or 6 in Sham group, *n*=7 or 8 in UUO group). (E-G) The mRNA levels of renal FN1, Collagen I and TGF-β1 were analyzed by qRT-PCR (*n*=4 or 6 in Sham group, *n*=8 in UUO group). (H-I) Representative images of Masson trichrome staining and Sirius Red staining of renal tissue (×200) after UIRI model. Scale bar, 50μm or 100μm. (J) Quantitative data of fibrosis area in UIRI model (*n*=5 in Sham group, *n*=7 or 8 in UIRI group). (K-M) Quantitative determination of renal Collagen I, Collagen III and α-SMA mRNA levels by qRT-PCR (*n*=5 or 6 in Sham group, *n*=7 or 8 in UIRI group). (N-O) Western blotting analysis of Collagen III levels (*n*=3 in Sham group, *n*=4 in UIRI group). Data are presented as mean ± SEM. Statistical analysis was performed using One-way ANOVA followed by Bonferroni. The P-values were shown in the figures. Abbreviations: UUO, unilateral ureteral obstruction; UIRI, unilateral ischemia/reperfusion injury; Vehi, vehicle.


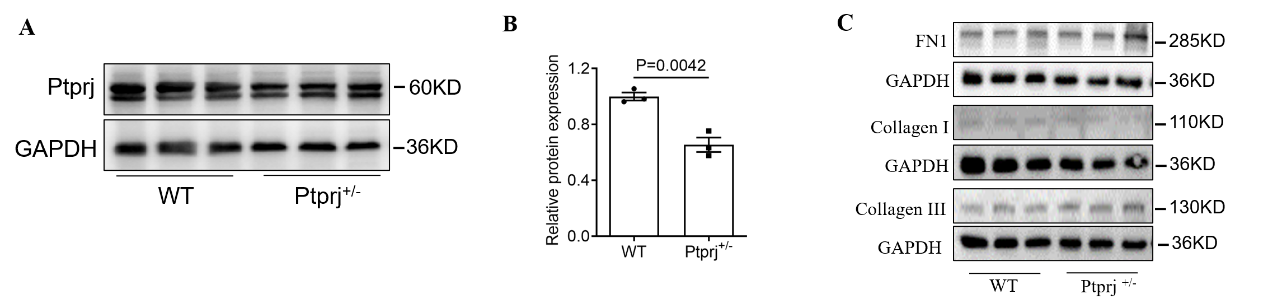


**Figure S4. Validation of Ptprj in heterozygous knockout mice.** (A-B) Western blotting analysis and quantitative data for Ptprj in WT and Ptprj^+/-^ mice (*n*=3 in each group). (C) Western blotting analysis and quantitative data for FN1, Collagen I and Collagen III in WT and Ptprj^+/-^ mice (*n*=3 in each group). Data are presented as mean ± SEM. Statistical analysis was performed using unpaired Two tailed Student's t-test. The P-value was shown in the figure.

**
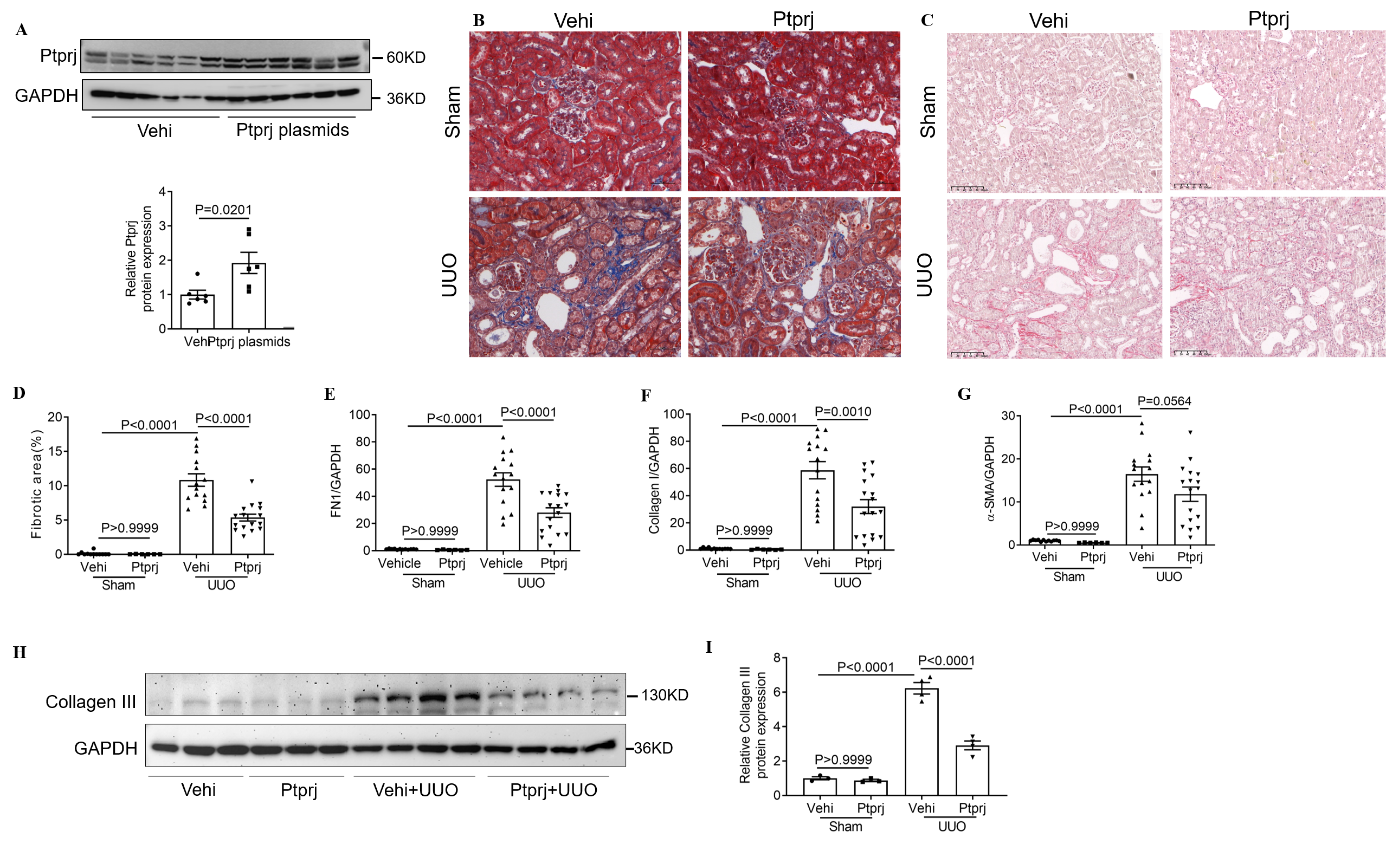
**

**Figure S5. Overexpression of Ptprj by high-throughput tail vein plasmid delivery in kidneys protected against UUO-induced renal fibrosis.** (A) Western blotting analysis of Ptprj in kidney after tail vein injection of Vehi or Ptprj plasmids (*n*=6). (B-C) Representative images of Masson trichrome and Sirius Red staining of renal tissue (×200) after UUO model. Scale bar, 50μm or 100μm. (D) Quantitative data of fibrosis area in UUO model (*n*=12 in Vehi+Sham group, *n*=6 in Ptprj+Sham group, *n*=14 or 16 in UUO group). (E-G) The mRNA levels of renal FN1, Collagen I and α-SMA were analyzed by qRT-PCR (*n*=12 in Vehi+Sham group, *n*=6 in Ptprj+Sham group, *n*=14 or 16 in UUO group). (H-I) Western blotting analysis of Collagen III levels (*n*=3 in Sham group, *n*=4 in UUO group). Data are presented as mean ± SEM. Data were statistically analyzed using unpaired Two-tailed Student's t-test (A) or One-way ANOVA followed by Bonferroni (D, E, F, G and I). The P-values were shown in the figures. Abbreviations: UUO, unilateral ureteral obstruction; Vehi, vehicle.

**
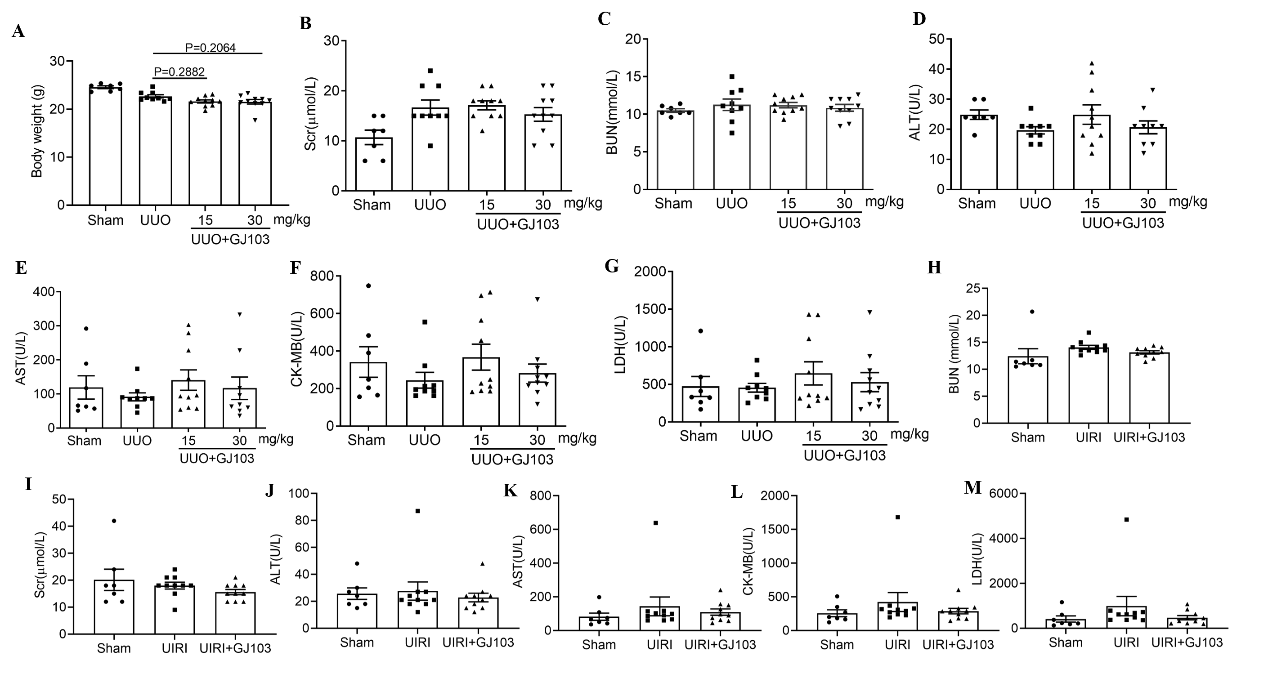
**

**Figure S6. The therapeutic doses of GJ103 had no obvious renal, hepatic, heart and systemic toxicity.** The mice were pre-treated with low dose (15 mg kg^-1^) or high dose (30 mg kg^-1^) of GJ103 before UUO surgery and then treated daily for 7 consecutive days and sacrificed. Body weight (A), serum concentrations of Scr (B), BUN (C), ALT (D), AST (E), CK-MB (F) and LDH (G) were detected. N=7-10. In Figure H-M, the mice were treated with high dose (30 mg kg^-1^) of GJ103 before UIRI surgery and then treated daily for 14 consecutive days and sacrificed. Serum concentrations of BUN (H), Scr (I), ALT (J), AST (K), CK-MB (L) and LDH (K) were detected. N=7-10. Data are presented as mean ± SEM. Statistical analysis was performed using One-way ANOVA followed by Bonferroni. The P-values were shown in the figures. Abbreviations: UUO, unilateral ureteral obstruction; UIRI, unilateral ischemia/reperfusion injury; BUN, blood urea nitrogen; Scr, serum creatinine; ALT, alanine transaminase; AST, aspartate transaminase; CK-MB, creatine kinase isoenzyme-MB; LDH, lactatedehydrogenase.


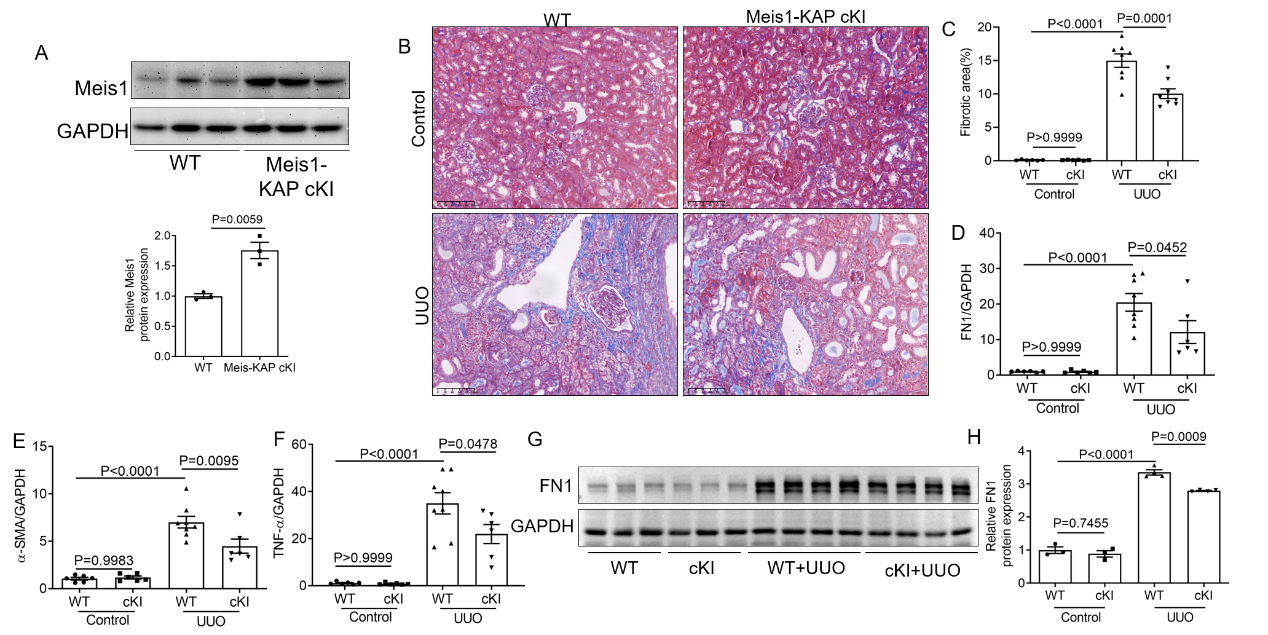


**Figure S7. Tubular-specific overexpression of Meis1 ameliorated renal fibrosis in UUO model.** (A) Western blotting analysis of Meis1 in tubular segment isolated from WT or Meis1-KAP cKI mice (*n*=3). (B) Representative images of Masson trichrome staining of renal tissue (×200) after UUO model. Scale bar, 100μm. (C) Quantitative data of fibrosis area in UUO model (*n*=6 in Control group, *n*=8 in UUO group). (D-F) The mRNA levels of renal FN1, α-SMA and TNF-α were analyzed by qRT-PCR (*n*=6 in Sham group, *n*=6 or 8 in UUO group). (G-H) Western blotting analysis of FN1 levels (*n*=3 in Sham group, *n*=4 in UUO group). Data are presented as mean ± SEM. Data were statistically analyzed using unpaired Two-tailed Student's t-test (A) or One-way ANOVA followed by Bonferroni (C, D, F, F and H). The P-values were shown in the figures. Abbreviations: UUO, unilateral ureteral obstruction; KAP, kidney androgen-regulated protein.


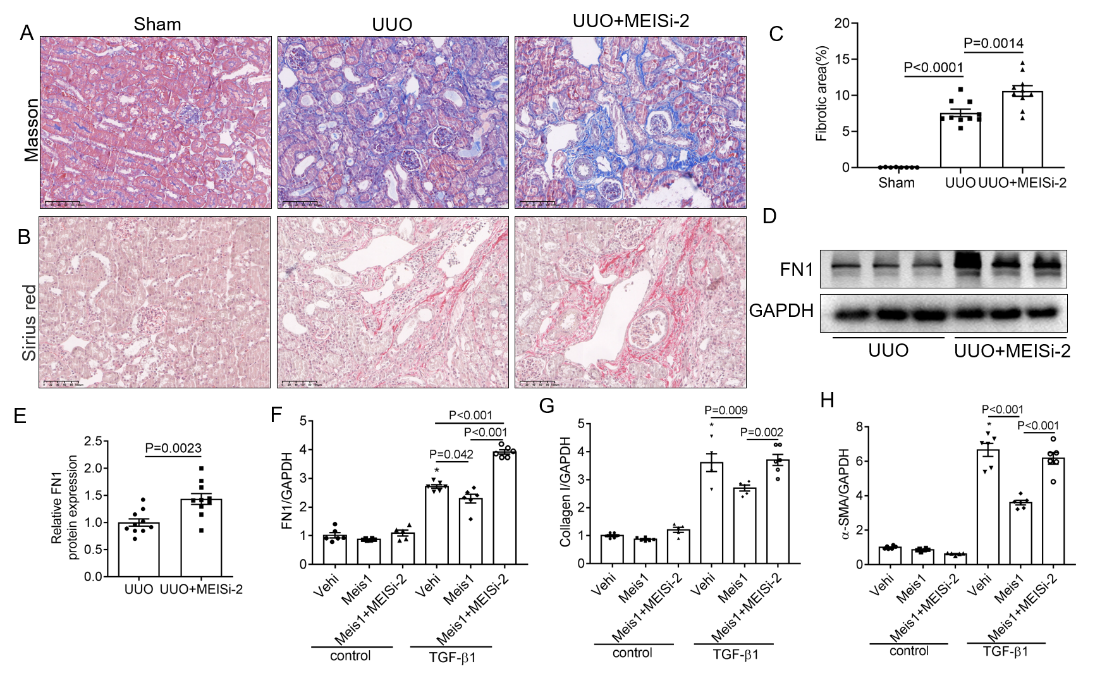


**Figure S8. Meis1 inhibitor deteriorated renal fibrosis after UUO model and promoted the activation of NRK-49F cells.** (A-B) Representative images of Masson trichrome staining and Sirius Red staining of renal tissue (×200) after UUO model. Scale bar, 100μm. (C) Quantitative data of fibrosis area in UUO model (*n*=8 in Sham group, *n*=10 in UUO and UUO+MEISi-2 group). (D-E) Western blotting analysis of FN1 levels (*n*=10 in UUO and UUO+MEISi-2 group). (F-H) NRK-49F cells were transfected with vehi or Meis1 plasmids, and then treated with MEISi-2 (1μM) for 1h, following TGF-β1 (10ng mL^-1^) treatment for 24h. qRT-PCR analysis of FN1, Collagen I and α-SMA mRNA expressions (*n*=5 or 6 in each group). Data are presented as mean ± SEM. Data were statistically analyzed using unpaired Two-tailed Student's t-test (E) or One-way ANOVA followed by Bonferroni (C, F, G and H). The P-values were shown in the figures. Abbreviations: UUO, unilateral ureteral obstruction; TGF-β1, transforming growth factor-β1; Vehi, vehicle.

**Table S1. The basic information and diagnosis of CKD patients**

| **Sex** | | **Age(yr)** | **Pathological**  **diagnosis** | **Scr**  **（μmol/L）** | **BUN（mmol/L）** | **Proteinuria**  **(g/24 h)** |
| --- | --- | --- | --- | --- | --- | --- |
| Male | 13.5 | | IgAN | 175.0 | 20.62 | 0.56 |
| Female | 12.0 | | IgAN | 39.0 | 3.92 | 2.73 |
| Female | 14.2 | | LN | 46.2 | 4.48 | 0.95 |
| Female | 10.8 | | AAV | 311.2 | 21.53 | 2.72 |
| Male | 13.6 | | HSPN IIIa | 91.0 | 4.00 | 2.91 |
| Male | 11.1 | | IgAN | 64.8 | 7.13 | 1.35 |
| Male | 8.0 | | Tubulointerstitial nephropathy | 387.0 | 20.10 | - |
| Female | 5.4 | | FSGS | 84.0 | 104.60 | 1.33 |
| Female | 11.8 | | FSGS | 74.0 | 7.90 | 2.48 |
| Female | 18.0 | | FSGS | 72.0 | 4.60 | 0.61 |
| Male | 4.1 | | FSGS | 20.0 | 7.60 | 2.25 |
| Male | 13.3 | | FSGS | 228.9 | 26.81 | 3.38 |
| Male | 5.4 | | FSGS | 48.0 | 8.00 | 2.16 |
| Male | 14.8 | | Podocytopathy | 75.0 | 7.30 | 7.17 |
| Female | 14.6 | | AAV | 659.0 | 25.05 | 2.87 |
| Male | 16.7 | | FSGS | 64.4 | 3.24 | 7.02 |
| Male | 1.3 | | Sclerosing glomerulonephritis | 75.7 | 12.24 | - |
| Male | 46 | | IgAN | 79.9 | 5.25 | 0.14 |
| Female | 35 | | IgAN | 47.1 | 4.36 | 0.27 |
| Male | 27 | | FSGS | 80.6 | 6.62 | 0.07 |
| Male | 55 | | IgAN | 127.9 | 7.38 | 0.43 |
| Male | 36 | | IgAN | 161.2 | 13.05 | 18.37 |
| Female | 61 | | IgAN | 88.3 | 8.77 | 0.52 |
| Male | 50 | | IgAN | 142.0 | 7.17 | 3.48 |
| Male | 31 | | IgAN | 536.0 | 21.36 | 3.09 |
| Female | 57 | | DN | 206.7 | 14.23 | 4.99 |

**Table S2. The sequences of the primers used in the study**

| Primer Name | Primer Sequence 5＇-3＇ |
| --- | --- |
| Mouse-GAPDH | F：ACAACTTTGGTATCGTGGAAGG  R：GCCATCACGCCACAGTTTC |
| Mouse-Collagen Ⅰ | F：GAGGGCCAAGACGAAGACATC  R：CAGATCACGTCATCGCACAAC |
| Mouse-Collagen Ⅲ | F：GGAGCTGGCTACTTCTCGC  R：GGGAACATCCTCCTTCAACAG |
| Mouse-α-SMA | F：ACCTTTGGCTTGGCTTGTC  R：CGGACAGGAATTGAAGCGGAA |
| Mouse-Fibronectin | F：ATGTGGACCCCTCCTGATAGT  R：GCCCAGTGATTTCAGCAAAGG |
| Mouse-TGF-β1 | F：CTCCCGTGGCTTCTAGTGC  R：GCCTTAGTTTGGACAGGATCTG |
| Mouse-Fsp1 | F：TCCACAAATACTCAGGCAAAGAG  R：GCAGCTCCCTGGTCAGTAG |
| Mouse-Ptprj | F：GACTCAGGCGCTTCAGAATGT  R：TTGTTCAAGGTCTCATTGGTTGT |
| Mouse-Meis1 | F：GCAAAGTATGCCAGGGGAGTA  R：TCCTGTGTTAAGAACCGAGGG |
| Rat-GAPDH | F：CGGCAACTTCAACG GCACAGTCA  R：GGTTTCTCCAGGCGGCATG TCA |
| Rat-Collagen Ⅰ | F：GTGCTCCTGGTATTGCTGGT  R：GACCTTGAACTCCAGCAGGG |
| Rat-FN1 | F：TGGGACTGTACCTGCATTGG  R：CCCAGCAGCGTGATCAAAAC |
| Rat-Collagen III | F：CACCCCTCTCTTATTTTGGCAC  R：AGACTCATAGGACTGACCAAGGTAGTT |
| Rat-α-SMA | F：GCCGAGATCTCACCGACTAC  R：GAAGCGTTCATTCCCGATGG |
| Rat-Meis1 | F：ACACAGTGGGGATAACAGCA  R：GCCACGCCCTCATGATATTG |
| Rat-Ptprj | F：TCCAAATGCCCTGTCTGGTC  R：TTTTAGGGGCGGGTCCTTTC |

**Table S3. Sequences of ChIP primers**

| Primer Name | Primer Sequence 5＇-3＇ |
| --- | --- |
| -1575~-1569 | F：TCGCACTCCTGACCTCAAG  R：GGAGGCCGATACAAGGATGG |
| -1011~-1005 | F：GAGGGCCAAGACGAAGACATC  R：CAGATCACGTCATCGCACAAC |
| -512~-506 | F：ATGCGTCTCCAGAGTTCACA  R：AGAGCCCAGTACAAGCCAG |
| -434~-430 | F：ATCCCTGGACTGGCTTGTAC  R：GTGCGTGAACCCCGAAATT |
| +68~+74 | F：GCGGGAGGAAGCGATGAATA  R: GTCCCTGAGCGCGCA |
| GAPDH promoter | F: CTATCATCCGATCTGACCATCACG |
|  | R: CGCCCGACGCTTCTGGGAGTTGTA |
